# Number agreement processing in adolescents with and without developmental language disorder (DLD): evidence from event-related brain potentials

**DOI:** 10.1038/s41598-023-49121-1

**Published:** 2023-12-21

**Authors:** Émilie Courteau, Phaedra Royle, Karsten Steinhauer

**Affiliations:** 1 School of Speech Language Pathology and Audiology, Faculty of Medicine, University of Montreal, Montreal, QC Canada; 2 Department of Psychology and Neuroscience, Dalhousie University, Halifax, NS Canada; 3 Centre for Research On Brain, Language and Music (CRBLM), Montreal, QC Canada; 4 School of Communication Sciences and Disorders, Faculty of Medicine, McGill University, Montreal, QC Canada

**Keywords:** Human behaviour, Language

## Abstract

In morphologically richer languages, including French, one must learn the specific properties of number agreement in order to understand the language, and this learning process continues into adolescence. This study examined similarities and differences between French-speaking adolescents with and without developmental language disorder (DLD) when processing number agreement, and investigated how morpho-syntactic regularity affected language processing. Using event-related potentials (ERP) and only grammatical sentences with audio-visual mismatches, we studied ERP correlates to three types of number agreement: (1) regular determiner agreement in noun phrases, (2) regular subject-verb plural liaison, and (3) irregular subject-verb agreement. We also included a lexico-semantic mismatch condition to investigate lexico-semantic processing in our participants. 17 adolescents with DLD (M = 14.1 years) and 20 (pre)teens with typical language (TL, M = 12.2 years) participated in the study. Our results suggest three patterns. First, French-speaking teenagers without DLD are still consolidating their neurocognitive processing of morpho-syntactic number agreement and generally display ERP profiles typical of lower language proficiency than adult native speakers. Second, differences in morphosyntactic processing between teenagers with and without DLD seem to be limited to rule-based (regular) number agreement. Third, there is little evidence for corresponding differences in lexico-semantic processing.

## Introduction

To understand language, and more precisely, number agreement, children need to learn how to process the different forms a word may take, a branch of linguistics called inflectional morphology^1^ or morphosyntax. Children must process ruled-governed regular inflected words as found in subject-verb number agreement (e.g., 3rd singular *she sings*). In morphologically richer languages, including French, children need to learn the specific properties of regular number agreement such as subject-verb plural liaison (e.g., singular *elle achète* [εlaʃεt] ‘she_3sg_ buys’, plural *elles achètent* [εl**z**aʃεt] ‘they_3pl_ buy’) and determiner agreement paradigms in noun phrases (NP: e.g., le/la/les [lə/la/lε] “the_M.SG/F.SG/PL_”), versus irregular verb singular and plural forms (e.g., singular *il pond* [ilpɔ͂] ‘he lays_3sg_ (eggs)’, plural *ils pondent* [ilpɔ͂**d**] ‘they lay_3pl_’). Note that pronouns in these conditions do not bear any overt number marking due to lack of liaison contexts.

Developmental language disorder (DLD) is a neurodevelopmental disorder that interferes with language acquisition and affects between 3.7^2^ and 7.6%^3^ of the population, depending on the age of the individual and the criteria used to study prevalence. According to many theories, children with DLD are impaired in their ability to learn rule-governed morphosyntax. One such theory, Ullman’s Procedural deficit hypothesis (PDH)^4,5^, states that procedural memory underpins such rule-governed aspects of language (which are typically reflected by anterior negativities and P600 components in event-related potentials [ERP] studies, see below), and that brain structures abnormalities supporting this memory system can be found in DLD. In contrast, declarative memory is responsible, among other things, for stored information in the mental lexicon, i.e., lexico-semantics, and unpredictable word forms such as irregular verbs (all of which are associated with N400 ERP effects, see below). Declarative memory is expected to be preserved in DLD. While most research has focused on oral production in young children with DLD to examine how they master agreement, only few studies have examined how their brains process regular and irregular agreement, and none in French.

This gap in the literature is a concern because it is still unclear whether agreement deficits are a feature of DLD in French. Indeed, while agreement deficits have been observed in many languages including English^6^, Leonard (*ibid*) suggests that children speaking Romance languages (including French) do not show the severe deficits in number agreement that are found in English-speaking children. Furthermore, mixed results were found on grammatical deficits in French DLD. Using psycholinguistic tasks, Elin Thordardottir et al. (^7^, see also^8^) found that grammatical deficits in French-speaking preschoolers with DLD were not a particular weakness compared to their lexical-semantic abilities, whereas Courteau et al.^9^ did find this to be the case in teenagers, with a task assessing irregular verb production.

The ERP technique allows us to track cognitive processes underlying language, enabling us to understand when and how different linguistic operations unfold over time^10^. ERPs are well suited to the study of agreement because they allow us to test comprehension beyond behavioural scores on psycholinguistic tasks. Using electrode caps, the electroencephalogram records neural responses related to linguistic events, and from this signal ERPs are extracted. ERP components are described in terms of their polarity (negative or positive), timing (onset, peak latency, and duration of a brain wave), and topography (distribution on the scalp surface). Linguistic stimuli violating grammar rules (potentially related to procedural memory^4,5^) often result in a biphasic ERP pattern consisting of a frontal negativity around 300 ms that is followed by a posterior positive-going brain wave between 500 and 1000 ms (the P600 component). In contrast, linguistic anomalies involving stored lexical and semantic information tend to yield posterior negativities around 400 ms (N400 components)^10^.

In adults, ERP studies with auditory stimuli have shown that morphosyntactic violations initially elicit left or bilateral anterior negativities (ANs) associated with automatic grammar processing^10^ that can last up to 1200 ms after stimulus onset^11^. This ERP pattern has been observed in response to subject-verb number agreement processing, including when mismatches occurred between grammatical auditory sentences and pictures depicting incongruent subject number in French^12^. In Courteau et al. these types of audio-visual mismatches on regular and irregular subject-verb number agreement elicited sustained anterior negativities, while errors on regular determiner-noun number agreement elicited N400 components with a later onset and a more posterior scalp distribution. Negativities for morphosyntactic processing are usually followed by a positive-going brain wave, the P600 component, typically between 600 and 1000 ms after stimuli onset with a parietal scalp distribution^10^. The P600 has been associated with late, and probably controlled, sentence reanalyses and sentence categorization as incorrect^13,14^.

Biphasic patterns of a negativity followed by a positivity, reflecting morphosyntactic processing, can be found in children with typical language development (TL) starting at 8 years old, depending on the stimuli and experimental paradigm^15–17^. However, this biphasic pattern has not been observed in teens with DLD, and most ERP research has shown that teenagers with and without DLD exhibit distinct ERPs when processing number agreement. In response to auditory third person number agreement omissions in English in 16-year-olds (e.g., the boy often *cook), Haebig et al.^18^ found P600s in their TL group, and no effect for their DLD group, suggesting that participants with DLD did not process these errors. Weber-Fox et al.^16^ observed a biphasic pattern in a group of teenagers with TL but only a negativity for the DLD group for auditory third person number agreement omission errors. In response to regular subject-verb number errors in Italian, Cantiani et al.^19^ reported apparently similar ERP patterns for morphosyntactic processing (P600) in teenagers with and without DLD. However, ERP plots and running *t*-tests displayed in their figure suggest a smaller amplitude of the DLD group’s P600, which the authors did not discuss.

Steinhauer et al.^20,21^ propose a learning trajectory for morphosyntactic ERPs based on studies investigating second-language (L2) learners. They suggest that ERPs can reflect different stages of morphosyntactic proficiency, starting with the ‘novice’ level at which participants are still indifferent to morphosyntactic mismatches and may not elicit any ERP responses. Then, proficiency levels improve from very low to very high, as evidenced by a transition from N400s to small and delayed P600s, and finally to ‘nativelike’ biphasic ERPs (i.e., typically large P600s preceded by negativities). Applying this learning trajectory from L2 to first language (L1) acquisition, previous ERP work has shown that adolescents with TL exhibit either intermediate or very high proficiency levels (P600s^18^ or biphasic patterns^16^), depending on the stimuli, whereas adolescents with DLD exhibit ERPs characteristic for the novice level (no effect^18^) or very low proficiency levels (N400s^16^).

While no study so far has investigated irregular number agreement processing in DLD with ERPs (predicted by Ullman’s PDH to elicit N400-like components), many studies did examine lexico-semantic processing that is also reflected by N400s^22^. Lexico-semantic N400 effects are often elicited in priming paradigms or by semantically implausible sentences (e.g., *the cloud !laughed*). Similar N400s are found for crossmodal auditory-visual mismatches^23,24^, where an image (e.g., depicting a woman who is swimming) is presented concurrently with a mismatching auditory utterance, for instance *Chaque semaine, elle* !*chante ..* ‘Each week, she !sings…’^12^. ERP research using various lexico-semantic paradigms has consistently found that even children under two years elicit N400s similar to those found in adults^25^, pointing to early development of conceptual and lexical semantics. Importantly, similar N400s were found in participants with and without DLD^18,26^, but occasionally with a delayed onset, reduced duration, and different scalp distributions in the latter group^27,28^, possibly reflecting word frequency or other factors, which however have not been thoroughly investigated^29^.

There is a great need to investigate number agreement processing in French DLD. Indeed, it is still unclear whether agreement deficits might be present in DLD in morphologically richer Romance languages^6^ such as French, and mixed results have been found regarding whether grammar deficits in general are a reliable feature of DLD in French^7–9^. Our goal is to examine similarities and differences between teenagers with and without DLD when processing number agreement and to determine how number agreement regularity affects language processing in DLD. We studied the ERP correlates to three types of morphosyntactic number agreement: (1) regular determiner-noun agreement in NPs, (2) regular subject-verb agreement with plural liaison, and (3) irregular subject-verb agreement with consonant-final marking (i.e., the plural is signaled by the presence of a consonant at the end of the verb, while the singular is unmarked in the oral form). Based on previous ERP research and the PDH, we hypothesized that regular number agreement (1–2) would elicit distinct ERPs in participants with and without DLD^16,18^, as regular agreement is posited to be subserved by procedural memory, which is deficient in DLD^4,5^. We hypothesized that irregular agreement (3) would elicit similar ERPs in both groups, as it is expected to recruit lexico-semantic processes, subserved by declarative memory, which is preserved in DLD (*ibid*). Minor differences could emerge, however, because at the sentence level, participants may need to process abstract morpho-syntactic information between constituents^30^ (e.g., number agreement), which might engage the procedural memory system. To illustrate the (presumbably intact) lexico-semantic processes of our participants, we included a lexico-semantic mismatch condition that presented visually depicted actions and incongruent spoken verbs^12^. Lastly, we hypothesized that participants with DLD would exhibit ERP patterns reflecting lower levels of morphosyntactic proficiency when compared to the TL controls, based on the learning trajectory proposed by Steinhauer et al.^20,21^.

The current study uses a naturalistic visual-auditory paradigm that presents grammatical sentences and pictures to investigate morphosyntactic processing^12^, while avoiding a number of methodological shortcomings that are known to render ERP data difficult to interpret or even invalid, as described in Royle and Courteau^17^. Auditory-visual subject number mismatches (singular vs. plural) were created by varying the number of visually presented subjects and morphosyntactic number cues in the auditory stimuli.

## Methods

### Participants

A total of thirty-six pre-teens and teenagers with and without DLD participated in this study (they are a subset of ^9^). Their mother tongue, language of instruction and daily use was French. All participants’ parents gave written consent for their child’s participation prior to the first experimental session. All participants passed a hearing screening on the first day of assessment (500 Hz to 8000 Hz at 25 dB in at least one ear), had normal or corrected-to-normal vision, and had no history of major illnesses or prolonged hospitalization. Most of the participants were right-handed (*n* = 31), as assessed using the Edinburgh Handedness Inventory French adaptation^31^.

Of these 36 participants, 17 teenagers presented DLD (‘DLD group’), including 10 girls, aged between 12 and 15 years (M = 14.01; SD = 0.72). Most of them (*n* = 14) were recruited from a specialized private school for children and adolescents with learning disabilities in Montreal (Quebec, Canada) via an invitation letter sent by the school speech-language pathologist to parents of students who met selection criteria. Other participants were recruited from a parent's association for children with DLD. All participants had a documented history of DLD, with a complete speech-language pathologist’s language evaluation (including narrative and pragmatic domains) resulting in a diagnosis. All participants in the DLD group had been diagnosed before kindergarten or during the first year of primary school, and maintained significant functional impairments needing adaptations to succeed in school. These were for the most part accommodations in regular classes or enrolment in a special class, reflecting Bishop et al.'s ^32^ definition of DLD. Two participants had co-morbid dyspraxia, and nine co-morbid ADHD. These disorders do not preclude a DLD diagnosis (see *Statement 9*^32^). The percentage of participants with DLD and co-morbid ADHD in our study (53%) aligns with Mueller and Tomblin’s^33^ cohort study where co-morbidity with ADHD was observed in 40% to 49% of participants with DLD. A study by Redmond and colleagues^34^ shows that ADHD co-morbidity with DLD–and TL–does not increase children’s errors on language assessment tasks such as sentence recall. Additionally, while it has been documented that persons with ADHD may have procedural memory deficits^35^, a recent meta-analysis conducted by Sanjeevan et al.^36^ suggests that procedural learning is preserved in ADHD. Therefore, it is reasonable to assume that the inclusion of participants with co-morbid DLD and ADHD in our study did not interfere with our research objectives. Nevertheless, the dominant clinical profile of our DLD group was the presence of persistent language difficulties. Lastly, the group with DLD had significantly lower scores than the typical language group (TL) group (see Table 1) on the Word Classes Receptive task, which evaluates the ability to understand lexico-semantic class relationships, and Recalling sentences (both in CELF-IV^cnd-F^, French version,^37^). The Recalling sentence task, which marshals lexico-semantic, morphological, and syntactic domains, has been shown to discriminate between typical and disordered language development in English^38^ and in French^9,39^.

**Table 1:** Participant characteristics.

	DLD group (N = 17)		TL group (N = 19)		Brunner-Munzel tests		
	Mean	SD	Mean	SD	tbm	p-value	CLES
Age	14.01	0.72	12.48	1.92	3.11	0.004	0.75
School	7.53	0.51	6.16	1.92	2.05	0.052	0.69
Recal	56.76	7.59	68.74	8.89	6.83	< 0.0001	0.12
Word Rec	12.44	4.0	16.42	3.79	3.20	< 0.01	0.23
Corsi-F	5.56	1.55	5.55	1.76	0.12	0.91	0.51
Corsi-B	4.94	1.06	5.60	1.76	1.18	0.25	0.39
DMTS-1s	0.88	0.10	0.89	0.11	0.49	0.63	0.55
DMTS-5s	0.84	0.13	0.82	0.15	0.24	0.81	0.48

Comparisons between groups are expressed as the Brunner–Munzel statistic (tbm), a p-value and a common language effect size (CLES), indicating the probability of a random observation from the DLD group being larger than a random observation from the TL group, with 0.5 being at chance.

Chronological age (Age) and schooling (School) are expressed in years. Recalling sentences (Recal) and Word Classes Receptive (Word Rec) CELF-IV^cnd-F^ scores are untransformed, Corsi block scores reflect forward (Corsi-F) and backward (Corsi-B) spatial spans, and delayed match-to-sample represent the accuracy for 1 s (DMTS-1s) and 5 s (DMTS-5s) delays.

The remaining 19 participants with no history of language impairment (7 girls), aged between 8 and 14 years (M = 12.48; SD = 1.92) were included as controls in the typical language group (‘TL group’). A Chi-square test comparing the distributions of males and females in the two groups (10 F + 7 M in DLD; 7 F + 12 M in TL) did not find any significant differences (*X*^2^ (1, *N* = 36) = 1.7395, *p* = 0.1872). Their typical language developmental status was established via a questionnaire filled out during an interview with their parents, and confirmed by our linguistic and cognitive tasks. The groups did not differ on non-verbal abilities evaluated with tasks in the Cognitive Experiments IV v2 package of the Presentation^®^ software (Version 18.0, Neurobehavioral Systems, Inc., Berkeley, CA, https://www.neurobs.com). Non-verbal working memory was assessed with the forward and backward Corsi Block tasks^40^ and with a delayed match-to-sample task on non-verbal stimuli^41^ with delays of 1 or 5 s. Participant characteristics for both groups are presented in Table 1. To compare groups statistically, we used Brunner-Munzel tests^42^ as recommended by Rietveld and van Hout^43^ for group mean comparisons on skewed data with small sample sizes. Differences between groups were found in age (DLD > TL) and on the Recalling sentences and Word Classes tests (DLD < TL).

## Experimental task

Stimuli creation was inspired by the fLEX evaluation tool (fLEX: Multilingual assessment of inFlectional and LEXical processing,^44^ see Supplementary Materials Sect. 1 for details). The experimental tasks used in the present study are the same as those used in Courteau et al.’s study with French-speaking adults^12^, which we refer to for a detailed list of materials. Each participant listened to 300 spoken grammatical sentences paired with a picture that either matched (50%) or mismatched (50%) its morphosyntactic (*n* = 240) or semantic features (*n* = 60). Sentences began either with (1) a neutral context featuring a description of a general characteristic of the scene depicted in the picture (e.g., ‘each week’), or with (2) a subject context featuring a full NP that described the picture’s subject with lexical as well as morphosyntactic number agreement information (plural/singular, e.g., ‘the grandmother/s’). Following these contexts, verbs were presented within sentences containing third person pronouns, and a sentence continuation with a direct object NP, or prepositional phrase (PP, e.g., ‘in the public pool’) to avoid sentence-final effects in ERPs time-locked to verbs (Hagoort^45^, see also Stowe et al.^46^). See Tables 2, 3 and 4 for examples of sentences.

**Table 2:** Experimental lexico-semantic conditions and their corresponding visual stimulus.

Visual stimulus			
		Sample visual stimulus presented concurrently with auditory stimuli for lexico-semantic match (1a–b) and mismatch conditions (2a–b). Note that, in addition to the mismatch at the target verb (“swims” vs. “sings”), conditions 2a–b also include a second mismatch in the prepositional phrase (here: “public pool” vs. “concert hall”)	
Condition	Context	Sample auditory stimuli	
Semantic match	Neutral	(1a)	*Chaque semaine ǀ elle ****nage**** dans la piscine publique* ‘Each week ǀ she **swims** in the public pool’
	Subject	(1b)	*La grand-mère* ǀ *elle nage dans la piscine publique* ‘The grandmother ǀ she **swims** in the public pool’
Semantic mismatch	Neutral	(2a)	*Chaque semaine* ǀ *elle !****chante**** dans la salle de concert* ‘Each week ǀ she !sings at a concert hall’
	Subject	(2b)	*La grand-mère* ǀ *elle !****chante**** dans la salle de concert* ‘The grandmother ǀ she !sings at a concert hall’

Critical words are in bold.

Subject = overt subject NP; ! = lexico-semantic mismatch; ǀ = cross-splicing point.

**Table 3:** Experimental morphosyntactic conditions involving liaison (LIAIS) verbs and their corresponding visual stimuli.

Visual stimulus				
				Image A: sample visual stimulus for match (1a–b) and mismatch conditions (2c–d) in the singular. Image B: sample visual stimulus for match (2a–b) and mismatch conditions (1c–d) in the plural
Condition	Number	Context	Sample auditory stimuli	
Morphosyntax match	Singular	Neutral	(1a)	*À midi* ǀ *elle ****achète**** des bonbons au marchand* ‘At noon ǀ she **buys** candies from the **vendor**’
		Subject	(1b)	***La**** petite fille* ǀ *elle ****achète**** des bonbons au marchand* ‘**The** little girl ǀ she **buys** candies from the **vendor**’
	Plural	Neutral	(2a)	*À midi* ǀ *elles‿****achètent**** des bonbons au marchand* ‘At noon ǀ they **buy** candies from the **vendor**’
		Subject	(2b)	***Les**** petites filles* ǀ *elles‿****achètent**** des bonbons au marchand* ‘**The** little girls *ǀ* they **buy** candies from the **vendor**’
Morphosyntax mismatch	Singular	Neutral	(1c)	*À midi* ǀ elle *achète* des bonbons au marchand* ‘At noon ǀ she *buys candies from the **vendor’**
		Subject	(1d)	*La* petite fille* ǀ elle *achète* des bonbons au marchand* ‘*The little girl ǀ she *buys candies from the **vendor**’
	Plural	Neutral	(2c)	*À midi* ǀ elles‿*achètent* des bonbons au marchand* ‘At noon ǀ they *buy candies from the **vendor’**
		Subject	(2d)	*Les* petites filles* ǀ elles‿*achètent* des bonbons au marchand* ‘*The little girls *ǀ* they *buy candies from the **vendor**’

Critical words carrying morphosyntactic agreement number cues are in bold.

Subject = overt subject NP; * = number mismatch; ǀ = cross-splicing point; *‿* = *liaison.*

**Table 4:** Experimental morphosyntactic conditions involving consonant-final (CONS) verbs, and their corresponding visual stimuli. Critical words carrying agreement morphosyntactic number cues are in bold.

Visual stimulus				
				Image A: sample visual stimulus for match (1a–b) and mismatch conditions (2c–d). Image B: sample visual stimulus for match (2a–b) and mismatch conditions (1c–d)
Condition	Number	Context	Sample auditory stimuli	
Morphosyntax match	Singular	Neutral	(1a)	*Chaque printemps* ǀ *il ****pond**** dans le nid* ‘Each spring* ǀ* he **lays** in the nest’
		Subject	(1b)	***Le**** merle* ǀ *il ****pond**** dans le nid* ‘**The** blackbird* ǀ* he **lays** in the nest’
	Plural	Neutral	(2a)	*Chaque printemps* ǀ *ils ****pondent**** dans le nid* ‘Each spring* ǀ* they **lay** in the nest’
		Subject	(2a)	***Les**** merles* ǀ *ils ****pondent**** dans le nid* ‘**The** blackbirds* ǀ* they **lay** in the nest’
Morphosyntax mismatch	Singular	Neutral	(1c)	*Chaque printemps* ǀ il *pond* dans le nid* ‘Each spring* ǀ* he *lays in the nest’
		Subject	(1d)	*Le* merle* ǀ il *pond* dans le nid* ‘*The blackbird* ǀ* he *lays in the nest’
	Plural	Neutral	(2c)	*Chaque printemps* ǀ ils *pondent* dans le nid* ‘Each spring* ǀ* they *lay in the nest’
		Subject	(2d)	*L***es**** merles* ǀ ils *pondent* dans le nid* ‘*The blackbirds* ǀ* they *lay in the nest’

Subject = overt subject NP; * = number mismatch; ǀ = cross-splicing point.

Our experiment included three types of verbs, each one related to different mismatches. In the lexico-semantic conditions we used verbs that had a constant phonological form in singular and plural contexts (e.g., elle/s nage/nt [ɛlnaʒ] ‘she/they swim/s’). The lexico-semantic mismatches were created by presenting a verb (and its direct object NP or prepositional phrase) that did not match the depicted action and the general theme of the picture (e.g., the sound file described ‘she swims in the public pool’ and the image depicted ‘she sings at the concert venue’), as illustrated in Table 2.

Morphosyntactic conditions included two verb types with different morpho-phonological and morphosyntactic properties in the plural. First, verbs with regular subject-verb agreement signaled by the phoneme [z] resulting from liaison between the pronoun’s plural form (i.e., *elles/ils* ‘they._FEM/MASC_’ [ɛlz/ɪlz]) and the vowel onset of the following verb (liaison verbs, LIAIS, see Table 3). Second, irregular verbs where the plural is signaled by the presence of a consonant at the end of the verb (consonant-final verbs, CONS, see Table 4). In the morphosyntactic incorrect conditions, the mismatches were created by using incongruent number agreement between the auditory and the visual stimuli (e.g., the sound file described two girls buying candies and the image depicted one girl buying candies). Each sentence in these conditions (LIAIS or CONS) could include either one or two number agreement mismatches: in sentence-initial neutral contexts, the mismatches occurred only on the verb, while in sentences with subject NP contexts, the mismatches occurred both within the subject context, at the determiner, and on the verb. See Tables 3 and 4 for examples.

The experiment included 180 French verbs acquired before age 8 selected from the Manulex database^47^. All verbs were matched across types on lemma frequency (SEM: M = 1.54, SD = 0.78; CONS: M = 1.73, SD = 0.87; LIAIS: M = 1.45, SD = 0.87), age of emergence, and length (syllables and phonemes). Manulex and Lexique^48^ were consulted to ensure that all nouns, adverbs, prepositions and adjectives used were age-appropriate and frequent. See Supplementary Materials Sect. 1 for more details. Subject grammatical gender (feminine or masculine), as well as syllable length of context phrases and of full sentences were balanced across the three verb types. For each verb, two colour drawings, with either 1 or 2 agents, were created by a professional artist. Drawings had a constant visual complexity level, avoiding superfluous or distracting details, and emphasized the action and agents described.

Sentences were recorded by a professional French-Canadian actress who read all sentences in all contexts (i.e., neutral and subject NP sentences in the singular and plural). She clearly articulated the words with natural intonation while avoiding coarticulation. Auditory stimuli were recorded at 44.1 kHz in a sound attenuated booth using a Sony DAT recorder (PCM-M1, 1997). To ensure a constant voice amplitude, a sonometer was placed 10 cm in front of her mouth to monitor for deviations of ± 5Db. Using Praat software^49^, we spliced the sentence-initial contexts to ensure that the neutral context for any given verb was identical in its singular and plural version, and to provide identical contexts in lexico-semantic conditions. Thus 1200 sentences were created. These were distributed throughout 4 lists resulting in 300 sentences by list. Lists were created in a counterbalanced manner where half of the sentences had neutral contexts and the other half subject NP contexts, and half of the sentences were singular and the other half plural.

### EEG recording

EEG was recorded continuously with a sampling rate of 500 Hz from 32 cap-mounted electrodes (WaveGuard active shielded caps, ANT, Enschede, NL) placed in accordance with the international standard 10/20 system. Electrodes used for recording cover the frontal, parietal, temporal and occipital lobes were: FP1, FP2, F3, F4, F7, F8, Fz, C3, C4, Cz, P3, P4, Pz, T7, T8, P7, P8, O1, O2, and Oz. All impedances were maintained below 5 kΩ and checked prior to and after recording. EEG was amplified with an ANT Neuro eego^TM^sports amplifier referenced to the CPz electrode.

### Procedure

The protocol was approved by the University of Montreal Research Ethics Board for educational and psychology research (CERES), and all methods were performed in accordance with the relevant guidelines and regulations. EEG recording took place in a quiet room either at the participants’ school or at the *Language Acquisition and Processing Lab* (Dr. Royle director) at the University of Montreal. Upon arrival, participants provided their parent’s written signed consent and underwent the audiology screen after which they were fitted with an EEG cap. Participants sat at a desk about 40 cm from a computer screen for the duration of the EEG session (1 h). Sentences were presented in an alien learning paradigm where Euzabie, a friendly alien visiting Quebec, was sitting in a classroom, and had to practice her French by describing pictures in a workbook. The participants' task was to indicate, by button press, whether Euzabie had made a mistake or not. A story containing filler sentences, illustrations, and animations was interspersed throughout the experience to maintain interest and attention. Participants listened to the spoken sentences presented binaurally via insert earphones (ER-1 Insert Earphones, Etymotic Research), while images were presented on the computer screen. A participant-controlled break was scheduled after each experimental block of 30 sentences.

Participants were asked to listen to each sentence while considering all aspects of grammar and meaning, and to judge sentence acceptability as appropriately describing the simultaneously presented image, by pressing one of the two keys on a response keyboard: "acceptable" or "not acceptable". In order to avoid laterality effects, the “acceptable” button was randomly assigned to right or left sides. Response keys had a smiley or sad face sticker so that participants did not have to memorize them. Participants were instructed to minimize movement and keep their eyes open during stimulus presentation. Six practice trials were presented at the beginning of the experiment and were excluded from further analysis. At least one researcher or assistant was present throughout the session. EEG recording was monitored during the experiment, and participants received feedback on blinking and other body movements whenever necessary, to reduce artifacts. Each trial had the following structure: a fixation cross was displayed in the centre of the screen for 1000 ms before stimulus presentation. Then the picture appeared 500 ms before the spoken sentence onset and stayed on the centre of the screen until the sentence ended. After the sentence, a blank screen appeared for 1000 ms, then a response prompt (‘???’) appeared on the screen and remained until a button was pressed, followed by a fixation cross for 1000 ms and a blank screen for 1000 ms. After EEG recording, participants completed experimental and clinical language evaluation tasks with the first author or a research assistant. Participants attended two two-and-a-half-hour sessions on average, including two ERP recording sessions: only data from the second EEG recording session are reported here.

### ERP measures

EEG data were processed offline using MATLAB (MathWorks, Natick, MA, USA, v. R2018 B), EEGLAB (v. 2019.1)^50^, Fieldtrip^51^, and the ERPscope R package (http://github.com/aherbay/erpscope) to illustrate effects^52^. Raw data were re-referenced to linked mastoids and filtered using Kaiser low-pass (40 Hz) and high-pass (0.3 Hz) filters. Three participants with DLD had bridged electrodes: channel interpolations resolved these issues for two of them while one was excluded from further analyses. EEG signals contaminated with eye blinks were corrected using independent component analysis (ICA)^53^. Movements and other artifacts were rejected using a 150 μV criterion, and all uncontaminated trials were entered into the final analysis. Single-subject EEG waveforms per condition were averaged separately over 2400 ms epochs (-600 to 1800 ms), time-locked to the relevant critical word onset (bold words in Tables 2, 3, 4), and entered into grand-average ERPs. Following artifact rejection, we retained a similar number of trials for both groups in lexico-semantic and morphosyntactic conditions with an average of 10% rejected trials for all conditions (see Supplementary Material Sect.  2). All participants were retained since we rejected less then 35% of trials per participant, the suggested criterion being 50% for patient studies^54^. ERP components were quantified and statistically analyzed as the mean EEG signal voltage (in μVs) in representative time-windows based on three criteria: (1) we used adults’ ERP effects in the same experiment^12^ as a guideline to select time-windows. (2) Based on previous literature on ERP components for children and adolescents^17^, we expected ERP effects to have later onsets than adults for number mismatch conditions, and consequently time-windows to be delayed as compared to adults. (3) We occasionally used smaller time windows to detect latency-related differences between the groups.

### Analyses

In both ERP and acceptability judgment analyses, we compared the mismatch conditions with their corresponding match controls. For instance, in the singular number mismatch sub-condition, we compared (1) spoken sentences containing a subject noun phrase in singular (agent) that were presented along with a matching picture depicting one agent (match condition) to (2) the exact same spoken sentences but now presented with a similar picture depicting two agents (mismatch condition).

Accuracy data from acceptability judgments were analyzed using detection theory for grammaticality judgment, which provides an unbiased measure of sensitivity including the participant’s ability to discriminate match and mismatch conditions^55^. The *A-*score (A'-score, corrected version^56^) was chosen because the groups’ accuracy judgments were characterised by both low and high sensitivity depending on the conditions, resulting in logistic or rectangular distributions^57^. *A-*scores of 1 reflect perfect discrimination, and 0.5 chance levels. We performed a first ANOVA with two factors, including the three Verb types (lexico-semantic, CONS, LIAIS) and Group (DLD vs. TL). The second one targeted morphosyntactic conditions and included four factors: Context (neutral vs. subject), Number (singular, plural), Verb (CONS, LIAIS) and Group (DLD vs. TL). The score distribution for the acceptability judgment accuracy were heavily skewed. Thus, we used Aligned Rank Transform ANOVAs for R, a non-parametric approach to factorial ANOVA, which relies on a preprocessing step that aligns data before applying averaged ranks^58^, with *A-*scores as the dependant variable. We report the omega-squared (ω^2^) as effect size, which is interpreted as follows: < 0.02, very small; 0.02 ≥ ≤ 0.13, small; 0.13 ≥ ≤ 0.26, medium; ≥ 0.26, large^59^. Post-hoc comparisons for interaction decomposition were done using the Wilcoxon-Mann-Witney test, and we applied the Bonferroni correction for multiples comparisons. We report the *r* statistic for effect size, which varies from 0 to close to 1. The interpretation values for *r*^60^ are: 0.10–0.3 (small effect), 0.30 ≥ ≤ 0.5 (moderate effect) and ≥ 0.5 (large effect).

Statistical analyses for ERPs were performed using the *Easy analysis and factorial experiments visualization package* in R (Lawrence, MA. 2011, R package version 4.4-0 3). When degrees of freedom were above two, Greenhouse-Geisser corrections were applied to address potential violations of sphericity: in these cases, the original degrees of freedom and corrected probability levels are reported. In each time-window global ANOVAs were performed separately on midline and lateral electrodes, with Group as a between-subject factor and the other factors as within-subject ones. For the midline channels, the semantic condition included 4 factors: Context (neutral vs. subject NP), Group (DLD vs. TL), Condition (mismatch vs. match), and Electrode (Fz, Cz, Pz, and Oz). At lateral electrodes, the ANOVA included 6 factors: Context, Group, Condition, Hemisphere (right vs. left), Anteriority (frontal, central, and posterior electrodes), and Laterality (lateral vs. medial). For morphosyntactic mismatch conditions, the factor Number (singular vs. plural) was included for both analyses. An alpha of *p* < 0.05 was used for all statistical analyses.

Analyses of ERPs effects for number mismatches at sentence onset were done by combining LIAIS and CONS sentences, as the distinction between our two kinds of verbs did not affect ERPs at this early point of the sentence. We compared mismatch and match conditions for both subject (e.g., ‘the blackbird/s’) and neutral contexts (e.g., ‘each spring’), collapsed across singular and plural sub-conditions.

We limited analyses of ERPs for number mismatches on verbs to neutral context sentences only since these were the ones presenting a number cue for the first time at the verb-target. At sentence onset, LIAIS and CONS conditions were indistinguishable, but downstream at the target verb they differed. For LIAIS verbs, this information is available at pronoun offset (i.e., *elles/ils* ‘they._FEM/MASC_’ [ɛlz/ɪlz]), which is why ERPs in this condition are time-locked to pronoun onset to avoid ERP baseline issues, while for CONS verbs, the verb-agreement number cue is available only at the verb-final phoneme (e.g., *ils pondent* [ilpɔ͂d] ‘they lay’) and ERPs are analysed at verb onset. As in Courteau et al.^12^, plural and singular sub-conditions are analysed separately, since in number mismatches on verbs they represent two different kinds of conditions: omission for singular verbs and commission for plural verbs.

## Results

### Acceptability judgments

When comparing *A-*scores for all verb types, we found a significant main effect of Group (*F*(1,34) = 16.94, *p* < 0.001, ω^2^ = 0.31), of Verb (*F*(2,68) = 40.86, *p* < 0.001, ω^2^ = 0.53) and an interaction between both factors (*F*(2,68) = 7.95, *p* < 0.001, ω^2^ = 0.16). Interaction decomposition revealed that the TL group performed better than the DLD group in sentences containing mismatches on CONS (U = 48.5,* p* < 0.001*, r* = 0.58, TL: MED = 0.92, DLD: MED = 0.85) and LIAIS verbs (U = 76,* p* < 0.01, *r* = 0.45*,* TL: MED = 0.94, DLD: MED = 0.85), but that both groups performed similarly when rating lexico-semantic conditions (U = 120,* p* = 2.0, r = 0.22, TL: MED = 0.96, DLD: MED = 0.95).

When considering morphosyntactic conditions, we found a main effect of Group (*F*(1,34) = 14.09, *p* < 0.001, ω^2^ = 0.27), with the TL group (MED = 0.94) performing better than the DLD one (MED = 0.86). A significant main effect of Verb with a small effect size (*F*(1,34) = 8.97, *p* < 0.001, ω^2^ = 0.18) revealed that *A-*scores were slightly higher for both groups on LIAIS (MED = 0.93) than CONS verbs (MED = 0.89). We found a main effect of Context (*F*(1,34) = 26.79, *p* < 0.001, ω^2^ = 0.42), where sentences with NP-subject contexts (MED = 0.93) were better identified than neutral ones (MED = 0.87). A significant Group × Context interaction (*F*(1,34) = 10.61, *p* < 0.01, ω^2^ = 0.21) revealed that this pattern was less prominent in the TL group, with a small effect (TL: U = 2330,* p* < 0.05, *r* = 0.17, NP-subject MED = 0.95, neutral MED = 0.92) compared to the DLD group where this effect was moderate (U = 1224,* p* < 0.001, *r* = 0.40, NP-subject MED = 0.90, neutral MED = 0.76). Interestingly, the decomposition of a significant Group × Number interaction (*F*(1,34) = 7.67, *p* < 0.01, ω^2^ = 0.16) revealed no differences between plural and singular sentences in the TL group (U = 3023,* p* = 0.6, *r* = 0.05, plural: MED = 0.93, singular: MED = 0.94), while those with DLD performed more poorly on singular sentences than plural ones (U = 2834,* p* < 0.05, *r* = 0.20, singular: MED = 0.80, plural: MED = 0.88). See Supplementary Material Sect. 3 for figures illustrating the effects described above and for tables listing all *A-*scores and accuracy data.

### Lexico-semantic mismatches

**Figure 1:**
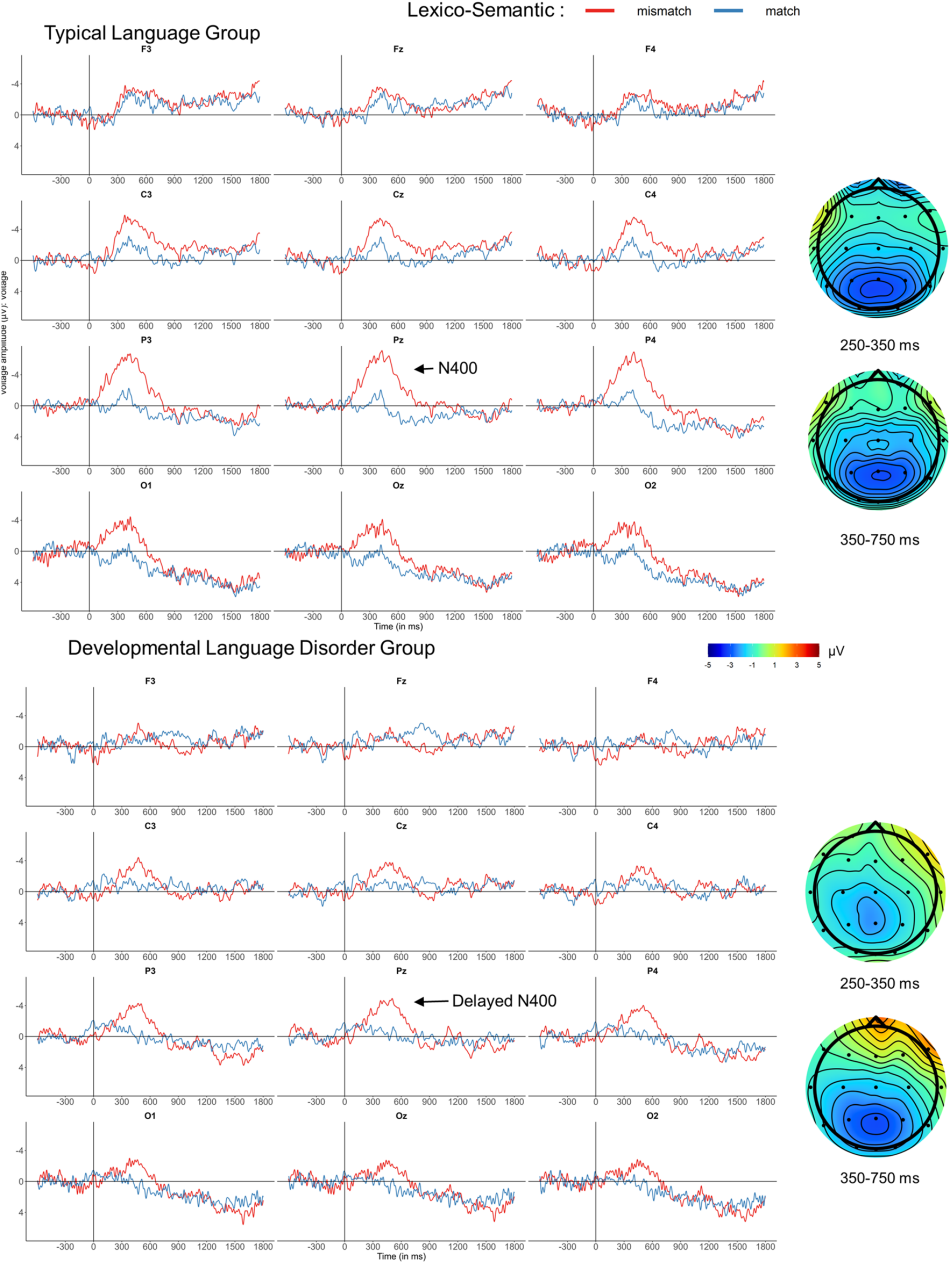
Effects elicited by the lexico-semantic mismatch conditions, collapsed across subject and neutral contexts. Grand-average ERPs for the TL (above) and DLD groups (below) are displayed at midline and eight lateral electrodes, as well as voltage maps illustrating difference waves, time-locked to critical verb onset using a baseline of − 600 to 0 ms. Verb onset is indicated by the vertical calibration bar. On average the verb ended 550 ms after onset; between 600 and 1900 ms participants heard a noun phrase or a prepositional phrase, which included a second lexico-semantic mismatch and ended the sentence. Compared with correct match conditions (blue line), lexico-semantic mismatches (red) elicited a large N400 that lasted from 250 to 750 ms for the TL group, and from 350 to 750 ms in the DLD group. Voltage maps represent difference waves (violation minus control), with negativities in blue and positivities in red.

At verb onset, lexico-semantic mismatches elicited broadly distributed N400-like negativities over centro-parietal electrodes in both groups, when compared to the match condition (Fig. 1). As in many auditory studies, the N400 had a longer duration beyond the classical 300–500 ms period typical for reading studies. As visual inspection suggested a slightly earlier onset for the TL group, we analyzed 2 time-windows: 250–350 ms for the N400 onset and 350–750 ms for the later portion of the N400, see Table 5.

**Table 5:** Global ANOVAs for lexico-semantic conditions at verb onset, for time-windows of interest.

	df	N400	
		250–350	350–750
Lateral electrodes			
Condition	(1, 33)		12.23**
Group × Condition	(1, 33)	6.08*	
TL: Condition	(1, 18)	25.73***	
Anteriority × Condition	(2, 66)		10.11**
Central: Condition	(1, 34)		16.02***
Posterior: Condition	(1, 34)		20.76***
Laterality × Condition	(1, 33)	7.58**	28.06***
Lateral: Condition	(1, 34)		4.82*
Medial: Condition	(1, 34)	4.29*	20.71***
Group × Hemisphere × Condition	(1, 33)	5.97*	
TL: Hemisphere × Condition	(1, 18)	6.05*	
TL: Right Hemisphere: Condition	(1,18)	23.27***	
Group × Laterality × Condition	(1, 33)	15.31***	
TL: Laterality × Condition	(1, 18)	28.39***	
TL: Medial: Condition	(1,18)	4.05†	
TL: Lateral: Condition	(1,18)	35.64***	
Group × Hemisphere × Laterality × Condition	(1, 33)	6.90**	
TL: Hemisphere × Laterality × Condition	(1,18)	6.50*	
TL: Left Hemisphere: Laterality × Condition	(1,18)	24.03***	
TL: Left Hemisphere: Medial: Condition	(1,18)	18.27***	
Midline electrodes			
Condition	(1, 33)	4.91*	22.54***
Group × Condition	(1, 33)	10.59*	
TL: Condition	(1, 18)	31.45***	
Electrode × Condition	(3, 99)	5.93**	13.47**
Cz: Condition	(1, 34)		19.97***
Pz: Condition	(1, 34)	9.86**	39.86***
Oz: Condition	(1, 34)	9.03**	16.97***

Only significant results are presented. *: *p* < 0.05, **: *p* < 0.01, and ***: *p* < 0.001.

Significant interactions between Condition and topographic factors were decomposed to identify distributional patterns. Global ANOVAs for the 250–350 ms time-window revealed Group × Condition interactions in both lateral and midline channels. Decomposing these interactions confirmed an earlier N400 onset for the TL group. Analyses in the 350–750 ms time-window yielded significant Condition effects in both lateral and midline channels supporting similar N400s for both groups, distributed from central to occipital regions.

### ERPs effects for number mismatches at sentence onset 

**Figure 2:**
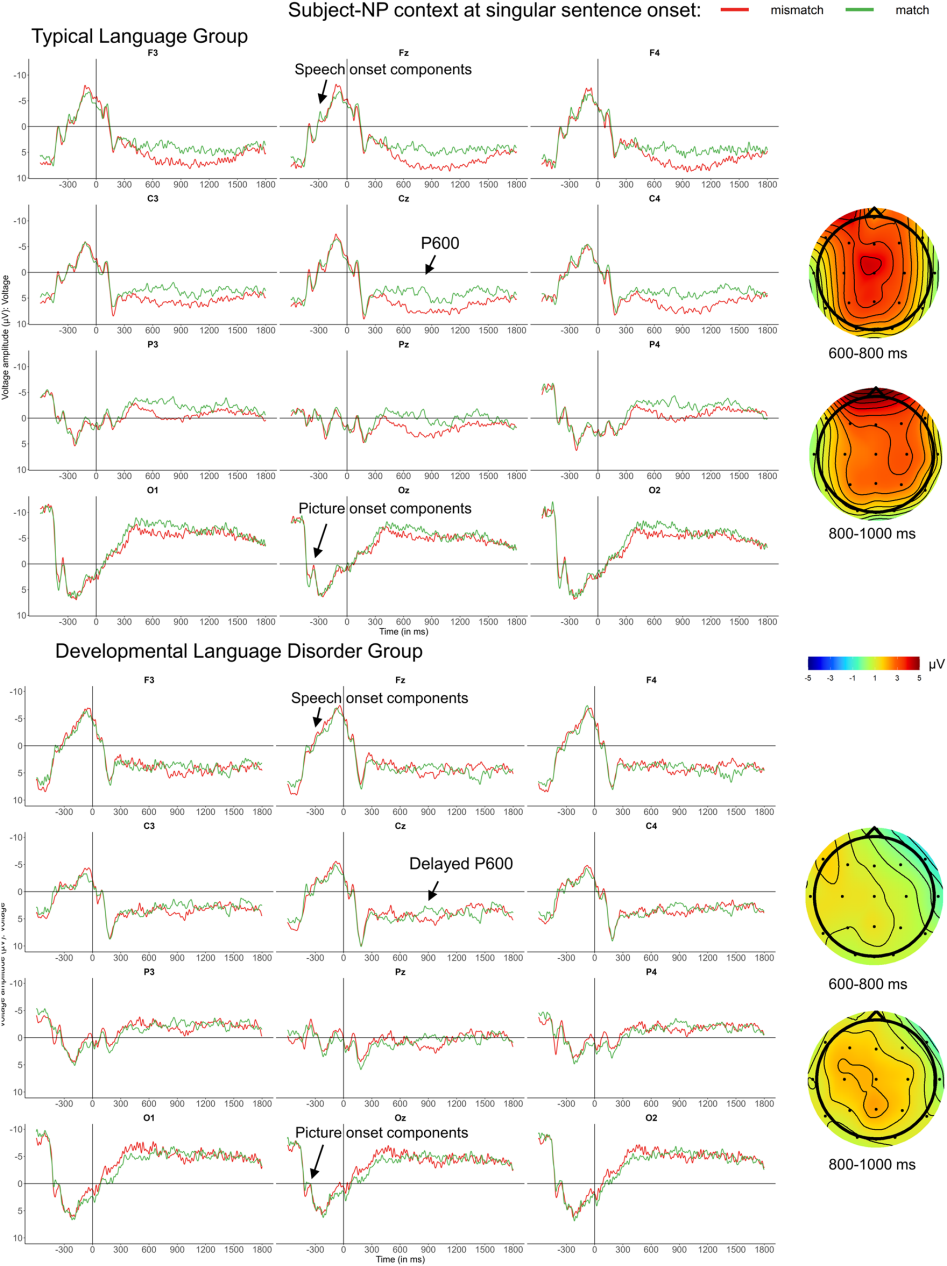
Singular subject NP context sub-conditions, at sentence onset: after virtually identical ERPs between −600 and 300 ms, which are dominated by visual and auditory onset components for pictures and spoken sentences, a broadly distributed positivity is elicited by singular subject context mismatches. Grand-average ERPs for the TL group (above) and the DLD group (below) are displayed at midline and eight lateral electrodes, as well as voltage maps illustrating the difference waves, time-locked to sentence onset (vertical bar) using a baseline of −600 to 0 ms. Compared to singular subject contexts in the match condition (green), mismatches elicited a broadly distributed P600-like positivity in both groups. In the TL group, this positivity began around 450 ms and was strong from 600 to 1000 ms, while in the DLD group, it was present only between 800 and 1000 ms. These distinct timelines for each group are illustrated in the voltage maps representing difference waves (mismatch minus match).

As determiner-noun agreement in French is regular and is supposed to rely on rule-based processing, the PDH would predict reduced effects in the DLD group. We initially compared mismatch and match conditions for both subject NP and neutral contexts, collapsed across singular and plural sub-conditions. As expected, neutral contexts did not differ, while the strongest number mismatch effects were carried by the singular conditions with subject NP contexts (illustrated in Fig. 2). Visual inspection of that contrast revealed a broadly distributed positivity in both groups for subject NP contexts, starting around 450 ms and reaching its maximum amplitude between 600 and 1000 ms for the TL group, whereas in the DLD group the effect was present only between 800 and 1000 ms. We ran Global ANOVAs on 600–800 ms and 800–1000 ms time-windows, as summarized in Table 6 (see Supplementary Material Sect.  4 for an additional figure including all conditions).

**Table 6:** Global ANOVAs for sentence onset conditions, for time-windows of interest.

	Df	P600-like positivity	
		600–800	800–1000
Lateral electrodes			
Context	(1,33)		6.57*
Hemi × Context	(1,33)		4.60*
Right Hemisphere: Context	(1,34)		9.77**
Group × Laterality × Context × Condition	(1,33)	6.65*	
Context × Number × Condition	(1,33)	5.00*	6.47*
SC: Number × Condition	(1,34)	9.74**	12.39***
SC: Singular: Condition	(1,34)	7.03**	14.83***
Lat × Context × Number × Condition	(1,33)		9.24**
Medial: Context × Number × Condition	(1,34)		7.70**
Medial: SC: Number × Condition	(1,34)		16.48***
Medial: SC: Singular: Condition	(1,34)		17.75***
Context × Laterality × Anteriority × Number × Condition	(2,66)	8.83**	
Midline electrodes			
Context	(1,33)		9.59**
Group × Context × Condition	(1,33)	5.21*	
Electrode × Context × Group × Condition	(3,99)	3.30*	
Context × Number × Condition	(1,33)	6.26*	8.08**
SC: Number × Condition	(1,34)	11.80***	12.30***
SC: Singular: Condition	(1,34)	8.97**	14.14***
Context × Number × Electrode × Condition	(3,99)	5.12**	
SC: Number × Elect × Condition	(3,102)	3.04*	

Only significant results are presented.

*SC* subject NP context.

*: *p* < 0.05, **: *p* < 0.01, and ***: *p* < 0.001.

In the Global ANOVAs including four conditions for the 600–800 ms and the 800–1000 ms time-windows, we found significant main effects of Context suggesting that both groups were able to distinguish between neutral and NP contexts. More importantly for the present study, we found significant effects of Condition (match vs. mismatch) shared by both groups for singular sentences in subject contexts, and the decomposition of the Context × Number × Condition interaction confirmed the broadly distributed P600-like positivity for the singular mismatch condition for both groups, as seen in Fig. 2. As illustrated by both the ERP plots and the topographic maps in Fig. 2, the time-course of this positivity was different in each group: for TL participants, the positivity started much earlier (around 450 ms) and seemed similar in both analyzed time-windows, whereas in the DLD group, the positivity emerges only between 800 and 1000 ms. To test the reliability of these group differences statistically, we ran an ANOVA comparing the two time-windows (i.e., including the additional factor Time-Window) at midline channels, on the mean amplitude of the positivity (i.e., the *difference wave* for mismatch minus match sentences). As expected, results indicated a main effect of Group (*F*(1,33) = 4.69, *p* < 0.05), which interacted with Time-Window (*F*(1,33) = 12.45, *p* < 0.001), confirming both a slower onset and a significantly smaller amplitude for the DLD group whose P600 peaked several hundred milliseconds later than in the TL group. See Supplementary Material Sect. 4 for decomposition of interactions.

### ERPs for number mismatches on verbs

#### ERPs for LIAIS plural conditions

**Figure 3:**
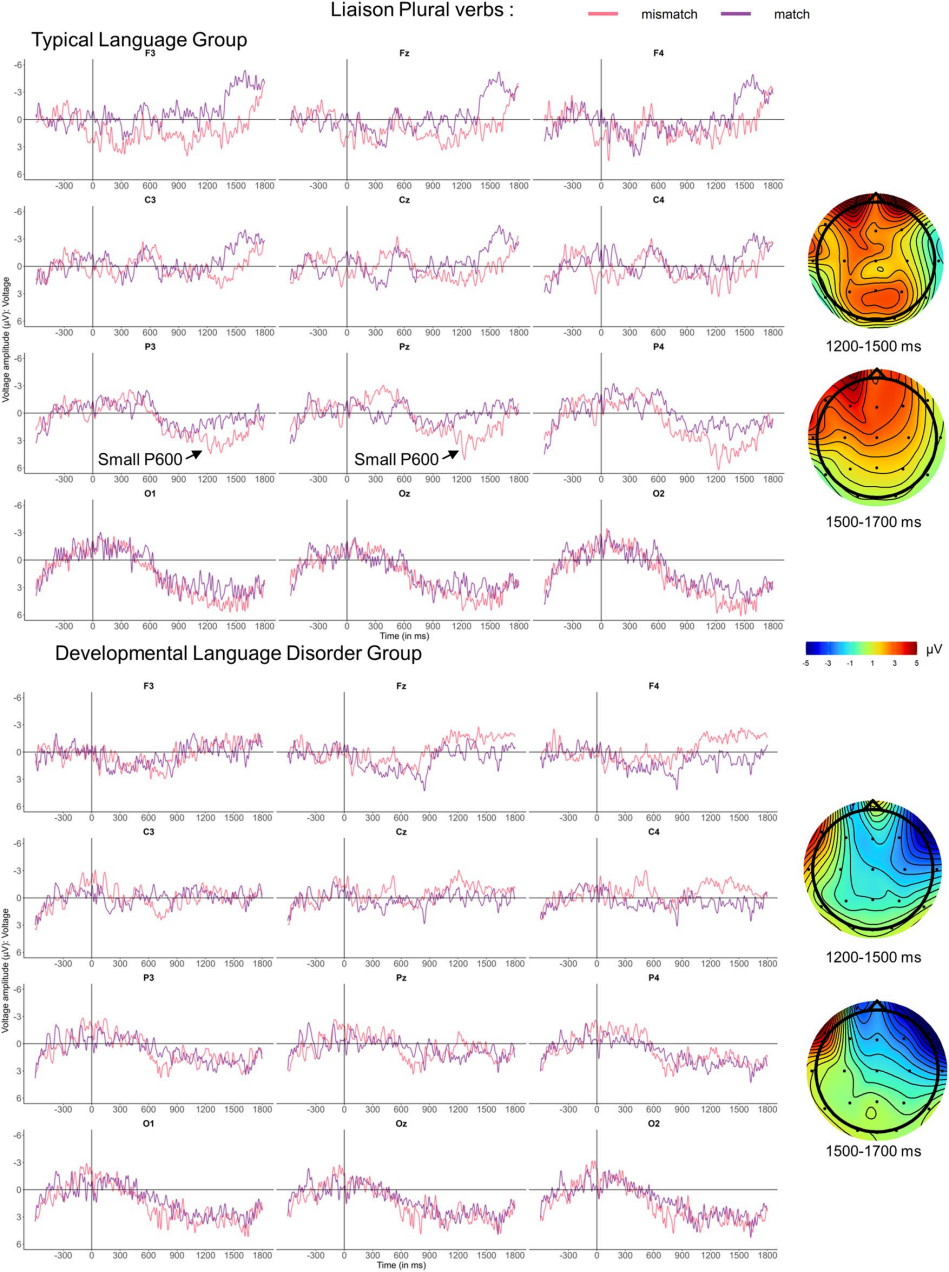
ERP number mismatch effects for plural LIAIS verbs in neutral contexts, time-locked to pronoun onset. Grand-average ERPs for the TL group (above) and the DLD group (below) are displayed at midline and eight lateral electrodes, as well as voltage maps illustrating difference waves time-locked to pronoun onset, using a baseline of −600 to 0 ms. Compared to the match condition (purple), the only reliable effects elicited by plural mismatches (pink) were found in the TL group. The TL group exhibited a small P600-like positivity from 1200 to 1500 ms, significant in medial electrodes and Pz, which shifted to a more frontal distribution from 1500 to 700 ms. Analyses in the 300–500 ms time-window revealed that the apparent N400-like negativity in the TL group did not reach significance. Note that we found significant effects in the 300–500 ms, 1200–1500 and 1500–1700 ms time-windows, in more lateral electrodes, that we refrained from interpreting because they appeared to be noise or eye-movement artefacts. See detailed explanations and an additional figure in Supplementary Material Sect. 5.

Liaison conditions are considered regular, such that the PDH would predict group differences. Visual inspection reveals that mismatches elicited an apparent small negativity between 300 and 500 ms at Cz in both groups (Fig. 3), which was non-significant (see Supplementary Material Sect. 5 for details). In the TL group, this was followed by a P600-like positivity from 1200 to 1500 ms in parietal and central electrodes that migrated to a more frontal distribution around 1500 ms. By contrast, in the DLD group a right-lateralized fronto-central negativity is observed from 1200 to 1700 ms. Thus, we selected 1200–1500 and 1500–1700 ms time-windows for statistical analyses, summarized in Table 7.

**Table 7:** Global ANOVAs for Liaison plural verbs at verb onset, for time-windows of interest.

	Df	Positivity	
		1200–1500	1500–1700
Lateral electrodes			
Hemisphere × Condition	(1,33)	5.75*	6.22*
Left Hemisphere: Condition	(1,34)	4.24*	5.13*
Anteriority × Hemisphere × Condition	(2,66)	8.13**	7.37**
Hemisphere × Laterality × Condition	(1,33)	4.45*	10.73**
Hemisphere × Anteriority × Laterality × Condition	(2,66)		8.73**
Group × Laterality × Condition	(1,33)	7.70**	
TL: Laterality × Condition	(1,18)	6.22*	
TL: Medial: Condition	(1,18)	5.21*	
Ant × Group × Condition	(2,66)		5.16*
Frontal: Group × Condition	(1,34)		5.40*
Frontal: TL: Condition	(1,18)		5.88*
Group × Anteriority × Hemisphere × Laterality × Condition	(2, 66)	4.02*	7.75**
Midline electrodes			
Group × Condition	(1,33)	4.69*	
Electrode × Group × Condition	(3,99)		3.63*
Fz: Group × Condition	(1,33)		4.69*
Fz: TL: Condition	(1,18)		4.60*

Only significant results are presented. *: *p* < 0.05, **: *p* < 0.01, and ***: *p* < 0.001.

For decomposition of interactions that we judged to be associated with noise and artefacts, see Supplementary Material Sect. 5.

Between 1200 and 1500 ms, the ANOVA yielded a significant Group × Laterality × Condition interaction which, when decomposed, confirmed the positivity for the TL group only, in medial lateral electrodes but not in midline channels. To confirm whether the apparent effect at Pz in the TL group (Fig. 3) was significant and part of the same positivity as observed at medial-parietal electrodes, we ran an additional ANOVA for the P3, P4, Pz channels with the factors Laterality (Medial vs. Midline) and Condition. We found a significant effect of Condition (*F*(1,18) = 6.14, *p* < 0.05), and no significant interaction, supporting our interpretation that the positivity was present in all parietal channels. In the 1500–1700 ms time-window, significant interactions involving Condition, Group and topographic factors confirmed that the TL group exhibited a somewhat left-lateralised positivity with a frontal distribution that was significantly different from the right-anterior negativity in the DLD group. In all time-windows, we found significant effects in extra-lateral channels reminiscent of artefacts, see Supplementary Material Sect. 5 for details.

#### ERPs for LIAIS singular conditions

Analyses did not point to any consistent ERP pattern for singular LIAIS verb mismatches in either group, see Supplementary Material Sect. 6 for a figure and results. Note that this is not surprising considering that LIAIS singular verb mismatches did not induce significant effects in the adult’s group either (see the discussion section in Courteau et al.^9^ for details on why this may be).

#### ERPs for CONS plural conditions 

**Figure 4:**
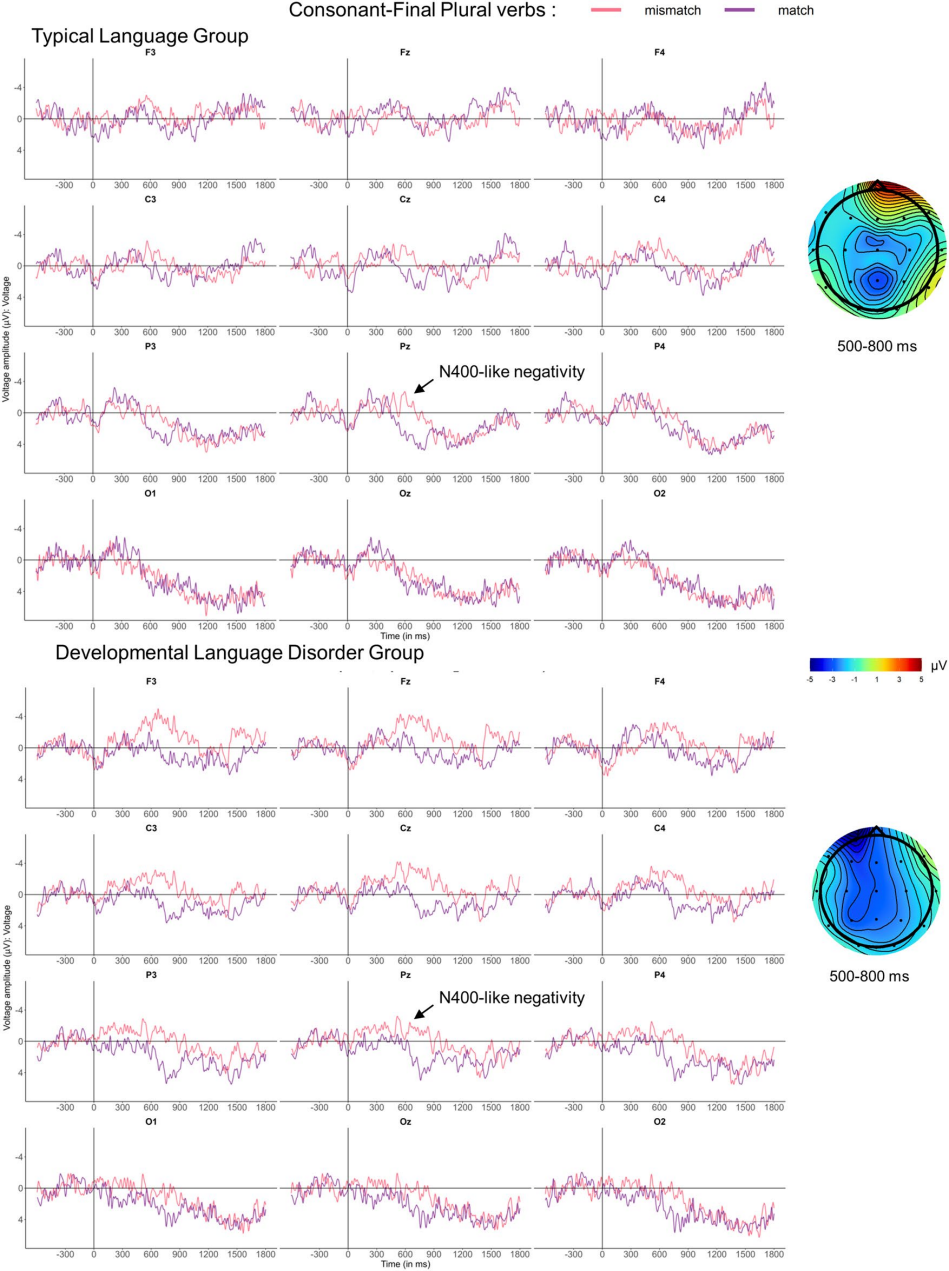
ERPs for number mismatch effects for plural CONS verbs in neutral contexts. Grand-average ERPs for the TL group (above) and the DLD group (below) are displayed at midline and eight lateral electrodes, as well as with voltage maps illustrating difference waves time-locked to CONS verb onset (vertical bar) using a baseline of −600 to 0 ms. In both groups, plural mismatches (pink) elicited a broadly distributed N400-like negativity between 500 and 800 ms compared to the match condition (purple). This time-window could be considered as rather late for an N400, but recall that in this condition the morphosyntactic cue revealing plural number is a verb-final consonant. On average, consonant onset was 275 ms following the verb’s onset, which explains the delay in this figure, as all ERPs were time-locked to verb onset. Apparent early (200–400 ms) and late differences (1200–1500 ms) did not reach significance.

Subject-verb agreement involving verb-final consonants are viewed as irregular and dependent on declarative memory like lexico-semantics. The PDH would predict no or only minor group differences. As seen in Fig. 4, visual inspection of the waveforms suggests that relative to match conditions, mismatches elicited small early negativities between 200 and 400 ms in both groups followed by an N400-like negativity between 500 and 800 ms. Between 1200 and 1500 ms, we observed a small centro-parietal positivity resembling a P600 in the TL group and a fronto-central negativity for the DLD group. However, as statistical analyses revealed no significant effects in either the early or the late time windows (200–400 ms, 1200–1500 ms), we will focus on the N400 (see Table 8).

**Table 8:** Global ANOVAs for consonant-final (CONS) plural verbs at verb onset from 500 to 800 ms.

	df	N400-like negativity
		500–800
Lateral electrodes		
Condition	(1,33)	4.17*
Laterality × Condition	(1,33)	9.47*
Lateral: Condition	(1,34)	
Medial: Condition	(1,34)	8.23**
Midline electrodes		
Condition	(1,33)	10.08**
Group	(1,33)	

Only significant results are presented. *: *p* < 0.05, **: *p* < 0.01, and ***: *p* < 0.001.

Analyses for the early effects in the 200–400 ms time-window did not reveal any significant effects or interactions involving Condition at either midline or lateral electrodes. Between 500 and 800 ms, a significant effect of Condition in both lateral and midline electrodes was found, thus confirming the presence of an N400-like negativity in both groups. The effect was most prominent at medial electrodes as compared to more lateral electrodes, as supported by a significant Condition × Laterality interaction. The analyses in the 1200–1500 ms time-window did not reveal significant effects or interactions.

#### ERPs for CONS singular conditions

**Figure 5:**
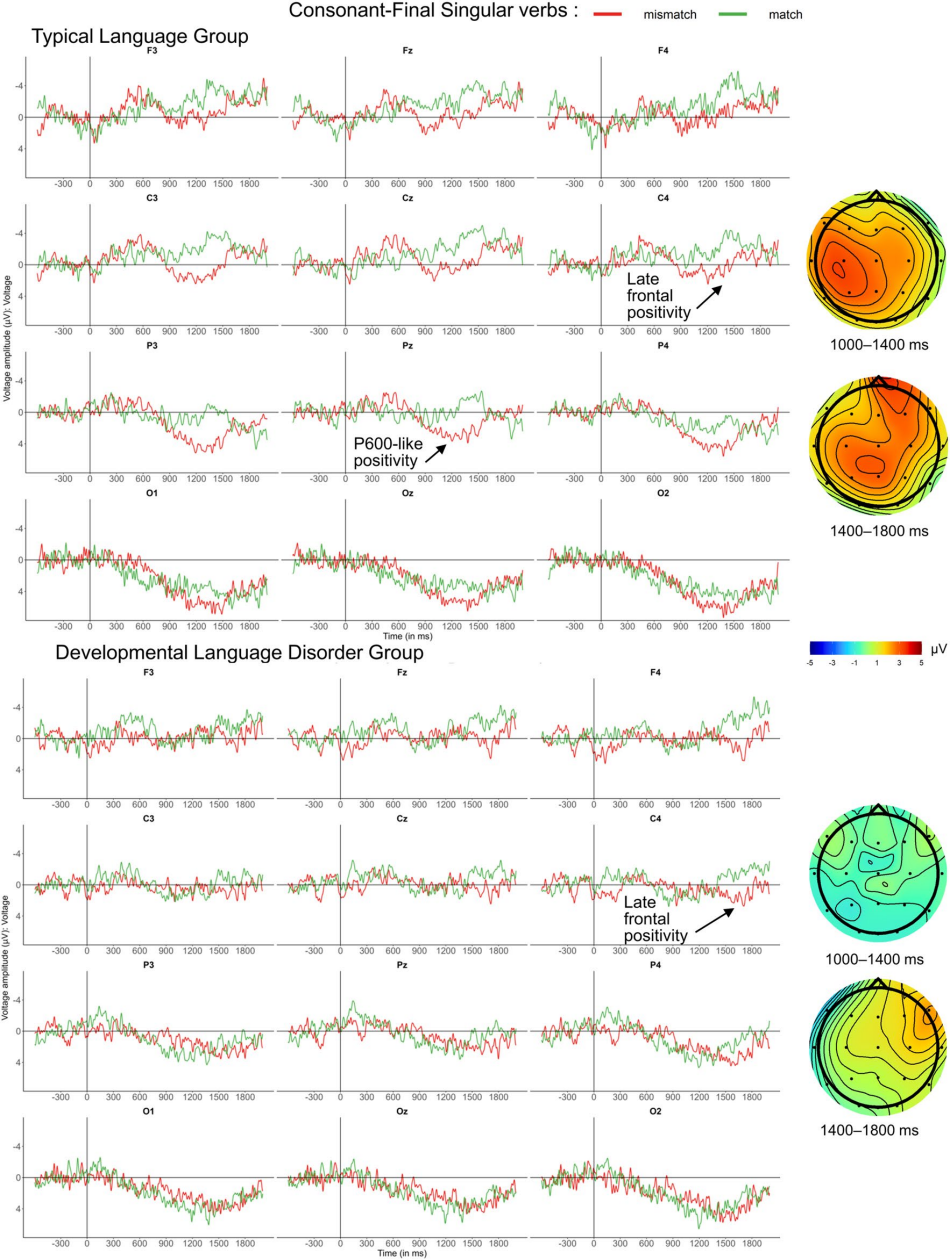
ERP number mismatch effects for singular CONS verbs in neutral contexts. Grand-average ERPs for the TL group (above) and the DLD group (below) are displayed at midline and eight lateral electrodes, as well as voltage maps illustrating difference waves, time-locked to CONS verb onset (vertical bar) using a baseline of −600 to 0 ms. Compared to the match condition (green), singular mismatches (red) elicited a P600-like positivity in the TL group from 1000 to 1400 ms. This could be considered late for a time-window reflecting a P600 but recall that participants could only have processed the singular information after the verb end, when omission of the plural number cue becomes apparent. In a somewhat later time window, mismatches elicited a right-lateralized frontal positivity in both groups from 1400 to 1800 ms. The apparent negativity elicited by the TL group between 500 and 700 ms did not reach significance.

As illustrated in Fig. 5, CONS singular mismatches in the TL group elicited an apparent small negativity (500–700 ms) in central electrodes near the midline which did not reach significance. This was followed by a large P600-like positivity with a posterior maximum from 1000 to 1400 ms, which spread to frontal channels between 1400 and 1800 ms. For the DLD group, mismatches elicited a fronto-central right-lateralized positivity (1400–1800 ms). The following time-windows were thus analyzed: 1000–1400 ms and 1400–1800 ms, see Table 9.

**Table 9:** Global ANOVAs for consonant-final singular verbs at verb onset, for time-windows of interest.

	df	P600-like positivity	Frontal positivity
		1000–1400	1400–1800
Lateral electrodes			
Condition			3.45*
Group × Condition	(1,33)	5.58*	
TL: Condition	(1,18)	5.85*	
Laterality × Condition	(1,33)		5.12*
Medial: Condition	(1,34)		5.17*
Anteriority × Hemisphere × Condition	(1,33)		4.96*
Frontal: Hemisphere × Condition	(1,34)		4.85*
Frontal: Right Hemisphere: Condition	(1,34)		11.2**
Group × Laterality × Hemisphere × Condition	(1,66)		5.40*
DLD: Laterality × Hemisphere × Condition	(1,15)		4.66*

Only significant results are presented. *: *p* < 0.05, **: *p* < 0.01, and ***: *p* < 0.001.

ANOVAs on the 1000–1400 ms time-window revealed significant interactions of Group and Condition in both lateral and midline electrodes. Decomposition of these interactions confirmed a broadly distributed P600-like positivity for the TL group only. In the 1400–1800 ms window, we found main effects of Condition in both lateral and midline electrodes and interactions between Condition and topographic factors showing that this frontal positivity was prominent in the right-hemisphere medial lateral electrodes in both groups. See additional analyses in Supplementary Material Sect. 7 confirming that the P600-like positivity and the late frontal positivity were two different components.

## Discussion 

The current study examined similarities and differences between French-speaking adolescents with and without DLD when processing number agreement, and investigated how regularity affected language processing. Using only grammatical sentences with audio-visual mismatches, we studied ERP correlates to lexico-semantic mismatches and three types of number agreement: (1) regular determiner-noun agreement in NPs, (2) regular subject-verb plural liaison, and (3) irregular subject-verb agreement. Following Ullman’s PDH^4,5^ and previous research^16,18,19^ , we expected to observe different ERPs between groups for regular conditions (1–2) assumed to rely on procedural memory, and similar ERPs for the irregular condition (3) and lexico-semantic mismatches, both of which are subserved by declarative memory. Overall, our results are in line with our hypotheses, with some caveats. We discuss how characteristics of the French language and our experimental design affected our results beyond morpho-syntactic regularity. Furthermore, in line with suggestions on how ERP profiles reflect learning trajectories, originally developed for L2 acquisition by Steinhauer et al.^20,21^, we found evidence that participants with DLD exhibited ERPs associated with lower levels of morphosyntactic processing proficiency when compared to the TL group.

As expected based on the PDH and previous research^18,26^, lexico-semantic mismatches on verbs elicited similar centro-paretial N400s in both groups, however with a short onset delay for the DLD group. Stimulus complexity is the best candidate to account for N400 onset delays in participants with DLD, as multiple lexico-semantic mismatches were present in the sentences. Again, consistent with the PDH, we found no difference between groups for acceptability judgments in lexico-semantic conditions, but a lower performance for the DLD group in morphosyntactic conditions.

For regular agreement (1–2), determiner agreement in NP at sentence onset elicited P600s, with differences in component onset: an early and large P600 was found in the TL group compared to a shorter and smaller one in the DLD group, which occurred with a delay of several hundred milliseconds. This pattern reflects substantial group differences predicted by the PDH, but also suggests that DLD teenagers still processed the mismatch to some extent. Subject-verb mismatches on regular LIAIS plural verbs elicited a small P600 in the TL group and no effect (or a right-frontal negativity) for the DLD one, also reflecting distinct ERPs in both groups as predicted by the PDH. Lastly, LIAIS singular conditions did not elicit reliable ERP effects for mismatches in either group. How can we explain the fact that three sentence conditions that were supposed to assess the same type of regular number agreement processing (assumed to rely on procedural memory^4,5^) elicited different ERPs in DLD and TL groups in two cases (determiners and LIAIS plural verbs) but *similar* ERPs in both groups in LIAIS singular conditions?

Firstly, the lack of group differences in the LIAIS singular condition simply reflects the absence of effects in both groups. In fact, even in healthy French adults, this condition did not elicit any ERP effects and resulted in poor performance^12^. The reasons have likely to do with a subtle sociolinguistic phenomenon in Quebec French that results in unreliable liaison production patterns, see Courteau et al.^12^ for details. Secondly, our observation that even in the LIAIS *plural* condition the DLD group did not show any indication of neurotypical P600s (whereas adults and TL teenagers did), is likely related to the subtle nature of this sociolinguistic phenomenon. As liaison agreement is supposed to rely on procedural memory, processing problems were predicted by the PDH. The fact that no reliable ERP effects were found in the DLD group at all may suggest that salient and consistent sociolinguistic cues may be necessary for teenagers with DLD to demonstrate at least some success in processing rule-based agreement. Indeed, this can be argued to be the case in the condition using sentence-initial determiners, as determiner agreement in French NPs—unlike liaison—is obligatory and consistently produced as a reliable and salient morphophonological cue^61^. In our opinion, it is this property of determiners that resulted in a small and late, but significant P600 in the DLD group. As one would expect, the presence of this determiner cue in addition to liaison markers (i.e., in LIAIS verb conditions with subject NP context) increased DLD participants’ accuracy in detecting mismatches: sentences with NP contexts were better identified than neutral ones, this effect being small in the TL group and moderate in the DLD one. In sum, it appears that while regular agreement conditions elicited group differences predicted by the PDH, saliency of cues also needs to be taken into consideration, with more salient cues being more likely to facilitate successful agreement processing in DLD, as reflected by both behavioural and ERP data.

For both lexico-semantic anomalies and irregular agreement (3) with CONS verbs, the PDH predicted similar ERPs in both groups as their processing is at least partly subserved by lexical retrieval from declarative memory. In contrast to rule-based processes that are primarily associated with biphasic AN -P600 patterns^10–12^, lexicon-based processes typically elicit N400 components, which are expected to be preserved in DLD^4,5^. Once again, our results are largely—but not entirely—in line with this hypothesis. Firstly, lexico-semantic mismatches elicited very similar N400s in both teenager groups, which moreover were largely identical with the ERP pattern previously found in adults^12^. Secondly, plural CONS verb mismatches elicited statistically indistinguishable N400 components in both groups, as predicted by our hypothesis^22^. Thirdly, subject-verb agreement involving singular CONS verb mismatches, revealed a considerable P600 onset delay of some 400 ms in the DLD group compared to TL controls. According to our discussion of group differences in regular agreement conditions above, such a delay would qualify as substantial—and thus as a pattern predicted by the PDH for regular rather than irregular morphosyntax, since no differences are expected between groups for irregular morphosyntax. Moreover, there is another observation that seems in conflict with the PDH prediction for irregulars, but is in line with PDH predictions for regulars: instead of an N400-like effect, we actually observed P600-like components in both groups. These ERP patterns, including the group differences, are clearly more typical for regular than irregular morphosyntactic mismatches. In our opinion, this is not a coincidence. The CONS contrast with singular verbs in both the match and the mismatch conditions is characterized by the *absence of* the irregular element (or consonant); it is thus reminiscent of an omission condition in regular morphosyntactic mismatches in English which usually only elicit P600s^62,63^. While plural CONS verbs used in subject-verb agreement mismatches arguably require the listener to ‘look up’ the irregular plural form in their mental lexicon to determine a commission error in the case of a singular subject NP, this additional lexical processing step linked to the N400 is less obvious in an omission scenario involving plural NPs and singular verbs. Moreover, previous work has not only shown that commission and omission conditions result in distinct ERP profiles but also that omissions are more subtle (i.e., less salient) and thus more difficult to detect (^62,63^, see also ^64^ for a saliency manipulation on the suffix). From this perspective, we would argue that the CONS condition with singular verbs might be better characterized as a regular omission condition. According to Ullman’s PDH^4^, this kind of “regular” agreement mismatch would reveal differences between groups because TL participants are expected to process regular agreement with an intact procedural memory, while those with DLD process it with an impaired one. In accordance with the PDH, we observed distinct ERP patterns, with the TL group showing a P600, whereas DLD showed a frontal positivity 400 ms later. Why even the TL group did not exhibit the expected adult biphasic pattern^12^ will be discussed below in the context of maturation and language proficiency. Note that P600s have been associated with “grammaticalization”^65^, i.e., learning processes underlying syntax and morpho-syntax, rather than with processes related to information stored in the lexicon (which are associated with the N400), although Ullman^35^ does not directly associate the P600 with procedural memory. Differences between groups are also supported by our behavioural data where sentence number played a role in the DLD participants’ performance only. They identified singular mismatches (omissions) less accurately than plural ones (commissions).

With respect to the role of rule-based versus irregular morpho-syntactic mismatches, our data are largely in line with the distinctions drawn by Ullman’s PDH^4^, in terms of both (1) the predicted ERP profiles (P600s for regulars reflecting “grammaticalization”^65^ and “syntactic processing difficulties”^35^ versus N400s for irregulars reflecting lexical processing in declarative memory) and (2) the presence of group differences between TL and DLD teenagers (predicted for regulars) versus their absence (predicted for irregulars). Overall, our data can be viewed as tentative support for the central PDH notion, according to which DLD may affect the procedural memory system more than the declarative memory system^4,5^.

 Regarding our second research question, namely whether proficiency-based trajectories observed in second language learners^20,21^ might also (a) apply to first language acquisition and (b) potentially point to additional delays in DLD, we also found preliminary support for these hypotheses. Our morphosyntactic conditions did not elicit mature biphasic ERP patterns in either group of teenagers, contrary to what was observed in adults in the same experimental paradigm ^12^. In Table 10 we present a summary of the elicited ERP patterns for morphosyntactic conditions for all groups, including adults.

**Table 10:** Summary of elicited ERPs for number agreement and lexico-semantic (mis)matches in French.

	Adolescents with DLD	Adolescents with TL	Adults^a^
Lexico-semantic mismatches	N400 (minor delay)	N400	N400 + additional negativities
**Regular number agreement**			
Determiner-noun agreement in NPs	Small + delayed P600	Large P600	N400-P600
Subject-verb plural liaison	No effect	Small P600	Early anterior negativity, N400-P600
Subject-verb singular liaison	No effect	No effect	No effect^a^
**Irregular number agreement**			
Consonant-final plural verbs	N400	N400	N400-P600
Consonant-final singular verbs (processed like English *regular* omission, see text)	Late frontal positivity	P600	Sustained anterior negativity, N400-P600

*DLD* developmental language disorder, *TL* typical language.

^a^For detailed ERP results of adults in the same experiment and a discussion on the absence of effect for the subject-verb singular liaison conditions, see Courteau et al.^12^.

In line with our first hypothesis (a) of a proficiency-based trajectory similar to L2 learners^20,21^, the data suggest that our participants with TL, aged 12 years old on average, are still maturing and learning how to process number agreement, which is coherent with previous literature on adolescents with TL^18,19^. In short, whereas adults typically display a biphasic ERP pattern of a P600 preceded by a negativity, TL teenagers typically only showed one ERP component per mismatch, which has previously been described as indicative of less proficient language processing in L2 acquisition^20,21^. As pointed out by one of our reviewers, reduced or absent ERP components may also be due to component overlap between N400 and P600^66,67^, but without additional experimental manipulations^68^, neither our current data nor previous ERP findings in L2 learners^20,21^ can unambiguously identify the specific underlying mechanism. Consistent with our second hypothesis in (b), the DLD group exhibited ERPs that usually reflect even lower levels of morphosyntactic processing in almost all conditions. Recall that these group differences were most prominent in conditions that can be described as involving rule-based (regular) morphosyntax (Table 10). In determiner agreement in sentence onset NPs, we found a delayed and smaller P600 for the DLD group–corresponding to a low-to-intermediate level of proficiency–in comparison to an earlier one in the TL group, reflecting an intermediate level. For LIAIS plural verb mismatches, the DLD group exhibited no effect, pointing to a novice level while the TL group exhibited a small P600, thus a low level of proficiency. For CONS singular verbs, which we have argued above can be viewed as a case of regular verb omission (as in English), we found a frontal positivity for the DLD group that was delayed by some 400 ms compared to the P600 observed in the TL group, corresponding respectively to a low and an intermediate level of morphosyntactic processing. Once again, adults showed the expected mature bi-phasic pattern, where the initial N400 likely reflected the mismatch detection, while the P600 was a correlate of morphosyntactic reanalysis.

Interestingly, and contrary to any general delay hypothesis for number agreement processing in French-speaking teenagers with DLD, plural CONS verb mismatches elicited indistinguishable N400s in both teenager groups. Compared to the biphasic N400-P600 profile in adults^12^, however, the monophasic N400 in teenagers seems to point to immature processing in both groups, suggesting that teenagers were still consolidating the plural form of these verbs. During an elicited oral production task using a subset of the verbs in our experiment, half of the participants in the TL group were still making between 5 and 10% of errors, and the DLD group made more than 20% error on average^9^. However, we suspect that this may not be the only reason for the elicitation of an N400 in response to these verb mismatches. Indeed, even if participants in the TL group were still consolidating CONS plural verb production, it would be surprising that these elicited more immature morphosyntactic processing than plural LIAIS verbs, which are produced in French oral language less than 20% of the time by adults^68^. Rather than signalling a very low level of morphosyntactic processing, another plausible explanation for these N400s is that they represent the expected pattern of lexical access for irregular plural verb forms, subserved by the declarative memory system^4,5^. Indeed, the other mismatch condition undoubtedly subserved by the declarative system, i.e., the lexico-semantic mismatch, also elicited very similar N400s in both teenager groups, with only a minor delay of 100 ms in the DLD group, while the adult group displayed a similar N400 followed by an additional negativity^12^.

Taken together, these data seem to suggest three patterns: (a) French-speaking teenagers with TL are still consolidating their neurocognitive processing of morpho-syntactic number agreement and generally display ERP profiles typical of lower language proficiency than adult native speakers. (b) There is little evidence for a corresponding delay in lexico-semantic processing. (c) Differences between TL and DLD teenagers seem to be limited to rule-based (regular) number agreement (compatible with Ullman’s PDH), where the ERP differences are compatible with a further developmental delay along the morpho-syntactic processing trajectory. Future studies could examine whether differences between TL and DLD participants persist into adulthood, to clarify whether morphosyntactic processing difficulties in DLD are due to a delay in the development of the procedural memory system or a specific deficit in this system.

There are two main limitations to this study. First, many participants of the DLD group had comorbid ADHD (9) or dyspraxia (2) disorders. Our study showed that participants in the DLD group had deficits in processing regular morphology, and thus procedural learning memory, compared to participants in the TL group. Given that ADHD and dyspraxia have been documented to be associated with motor deficits and thus possible impairments in procedural memory^35,36^, it is conceivable that these disorders may have contributed to our findings independently of DLD. However, it is important to note that exclusion of these participants would have been contrary to the DLD definition established by a consortium of researchers and clinicians, with DLD as a heterogeneous category that covers a wide range of deficits^32^. Inclusion of these participants with comorbid DLD disorders in our study favors generalization of our findings. Second, the participants with TL were slightly younger and had a wider age range than those with DLD. Nevertheless, TL participants exhibited more mature brain patterns than those with DLD. If we had included older participants with TL, we could have observed even greater differences between our groups in the morphosyntactic conditions.

## Data availability 

The preprocessed datasets analysed during the current study are available in the Files repository, https://osf.io/tkb3q/?view_only=ce73fae1b69c4d94b9759927f9d3eeef. The raw datasets are available from the corresponding author on request.

### Supplementary Information

Supplementary Information.
