# Supplementary material for: Number agreement processing in adolescents with and without developmental language disorder (DLD): evidence from event-related brain potentials
Source: Sci Rep. 2023 Dec 21;13:22836. doi: 10.1038/s41598-023-49121-1 (PMC10739941; doi:10.1038/s41598-023-49121-1)
Supplement: Supplementary file 1 — Supplementary Information. [file 41598_2023_49121_MOESM1_ESM.pdf]

Section 1. Stimulus selection

Stimuli were inspired by the fLEX evaluation tool, Task 4 (fLEX: Multilingual assessment of inFlectional and LEXical processing, [1]), where participants have to look at pictures while producing or listening to sentences involving singular and plural verb cues. A third of the pictures and verbs used in our experiment were taken from the original fLEX version, while two-thirds were developed following the same rules in order to have a sufficient number for an ERP experiment. Verb-selection criteria were: 1) number of phonemes and number of syllables, in Quebec French, in the 3rd person singular and 3rd person plural. Note that consonant-final verbs always had an additional phoneme in the plural; 2) lemma frequency (root plus inflected forms) and lexeme frequency (the inflected verb form) in spoken language from an oral language corpus [2] for 31 million words. Of the 180 verbs retained, 53 had one syllable, 110 had two and 17 had three. Their phonemic statistics are presented in Supplementary Table 1.

Supplementary Table 1. Verb distribution based on number of phonemes in the third person singular and plural present tense forms

Phonemes in the 3ps	#	Phonemes in the 3pp	#
2	23	2	10
3	28	3	33
4	49	4	42
5	54	5	53
6	15	6	28
7	10	7	10
8	1	8	4
Total	180	Total	180

Note. 3ps = third person singular; 3pp = third person plural

All verb categories–regular verbs with consonant onset (RegC), regular verbs with vowel onsets (LIAIS), and consonant final(CONS)–were matched item-by-item on these features. Some verbs were deliberately eliminated because of their low imageability (e.g., *essayer* ‘to try’) or difficulties illustrating them (e.g., *passer* ‘to pass’). T-tests with assumptions of equal, or unequal variance reveal unsurprisingly that LIAIS and CONS verbs differed in number of phonemes in the 3rd person plural, but also, unfortunately, in lexeme

frequency (in bold in Supplementary Tables 2 and 3). However, we decided to keep this selection of verbs as the differences were small and the standard deviations of the same order of magnitude.

Supplementary Table 2. Descriptive statistics on linguistic characteristics for each verb type

	LIAIS Mean	LIAIS SD	CONS Mean	CONS SD	RegC Mean	RegC SD
# syllables. 3ps	1.93	0.48	1.75	0.63	1.73	0,63
# syllables 3pp	1.93	0.48	1.75	0.63	1.73	0,63
# phonemes 3ps	4.40	1.21	3.95	1.40	4.42	1,39
# phonemes 3pp	**4.40**	1.21	**4.92**	1.43	4.42	1,39
Lemma frequency	1.45	0.87	1.73	0.87	1.54	0,78
Lexeme frequency	**0.85**	0.85	**1.17**	0.87	1.03	0,82

Note. 3ps = third person singular, 3pp = third person plural, LIAIS = regular verbs with vowel onset for liaison contexts, CONS = Consonant final verbs, RegC = Regular verbs with a consonant onset.

Supplementary Table 3. F- and T-tests for comparisons of each verb type on linguistic characteristics

	F-Test LIAIS vs CONS	T-Test LIAIS vs CONS	F-Test LIAIS vs RegC	T-Test LIAIS vs RegC	F-Test RegC vs CONS	T-Test RegC vs CONS
# syllables. 3ps	0.05	0.08	0.04	0.05	0.94	0.89
# syllables 3pp	0.05	0.08	0.04	0.05	0.94	0.89
# phonemes 3ps	0.28	0.06	0.28	0.94	0.99	0.07
# phonemes 3pp	0.20	**0.04**	0.28	0.94	0.85	0.06
Lemma frequency	0.99	0.08	0.40	0.57	0.41	0.21
Lexeme frequency	0.86	**0.04**	0.83	0.23	0.70	0.37

Note. 3ps = third person singular, 3pp = third person plural, LIAIS = regular verbs with vowel onset for liaison contexts, CONS = Consonant final verbs, RegC = Regular verbs with a consonant onset.

Section 2. Number of retained ERP trials per condition

Supplementary Table 4. Trials retained for each group in lexico-semantic and morphosyntactic conditions

**Groups**	**DLD**	**Typical development**
	Mean (SD)	Mean (SD)
Lexico-semantic	55/60 (6)	53/60 (6)
Number: Consonant-final verbs		
SINGULAR	27.6/30 (3.2)	26.9/30 (3.3)
PLURAL	28/30 (2.7)	26.2/30 (2.7)
Number: Liaison verbs		
SINGULAR	28.1/30 (2.3)	26.2/30 (3.1)
PLURAL	28.1/30 (2.3)	26.2/30 (3.6)

Section 3. Acceptability judgments

Supplementary Table 5. Judgment accuracy *A*-score averages (and standard deviations) for visual-auditory matching and mismatching trials in lexico-semantic verbs and number conditions for both consonant-final (CONS) and liaison verb (LIAIS) morphosyntactic conditions.

**Groups**	**DLD**	**Typical Language**
Condition	Mean (SD)	Mean (SD)
Lexico-semantic	0.94 (0.05)	0.96 (0.02)
Number: Consonant-final verbs	0.80 (0.10)	0.90 (0.07)
SINGULAR: NP-SUBJECT CONTEXT	0.81 (0.16)	0.90 (0.11)
SINGULAR: NEUTRAL CONTEXT	0.68 (0.16)	0.85 (0.13)
PLURAL: NP-SUBJECT CONTEXT	0.87 (0.14)	0.93 (0.04)
PLURAL: NEUTRAL CONTEXT	0.78 (0.15)	0.90 (0.10)
Number: Liaison verbs	0.83 (0.10)	0.91 (0.08)
SINGULAR: NP-SUBJECT CONTEXT	0.85 (0.15)	0.94 (0.06)
SINGULAR: NEUTRAL CONTEXT	0.73 (0.20)	0.88 (0.15)
PLURAL: NP-SUBJECT CONTEXT	0.89 (0.14)	0.94 (0.06)
PLURAL: NEUTRAL CONTEXT	0.79 (0.15)	0.89 (0.13)

Supplementary Table 6. Accuracy means (and standard deviations) for audio-visually matching and mismatching trials in lexico-semantic and number conditions for both consonant-final (CONS) and liaison (LIAIS) morphosyntactic conditions.

	DLD group		TL group	
Conditions	Match	Mismatch	Match	Mismatch
Semantics	84.7 (0.12) *30*	93.7 (0.06) *30*	88.8 (0.07) *30*	96.0 (0.04) *30*
NP-SUBJECT CONTEXT	84.7 (0.21) *15*	92.3 (0.09) *15*	93.7 (0.07) *15*	95.3 (0.06) *15*
NEUTRAL CONTEXT	84.7 (0.12) *15*	95.3 (0.08) *15*	84.0 (0.12) *15*	96.7 (0.04) *15*
Consonant-final verbs	85.1 (0.12) *60*	60.3 (0.18) *60*	91.2 (0.06) *60*	77.8 (0.15) *60*
SG: NP-SUBJECT CONTEXT	83.1 (0.24) *15*	67.5 (0.21) *15*	92.0 (0.01) *15*	77.3 (0.17) *15*
SG: NEUTRAL CONTEXT	85.1 (0.09) *15*	39.2 (0.22) *15*	91.6 (0.09) *15*	67.0 (0.29) *15*
PL: NP-SUBJECT CONTEXT	86.0 (0.23) *15*	78.0 (0.20) *15*	92.7 (0.06) *15*	85.3 (0.01) *15*
PL: NEUTRAL CONTEXT	86.3 (0.10) *15*	57.3 (0.25) *15*	88.3 (0.10) *15*	81.7 (0.13) *15*
Liaison verbs	83.2 (0.23) *60*	63.4 (0.19) *60*	93.4 (0.05) *60*	78.4 (0.19) *60*
SG: NP-SUBJECT CONTEXT	88.8 (0.12) *15*	69.7 (0.26) *15*	93.7 (0.07) *15*	85.3 (0.17) *15*
SG: NEUTRAL CONTEXT	91.0 (0.10) *15*	44.0 (0.28) *15*	93.0 (0.10) *15*	71.7 (0.22) *15*
PL: NP-SUBJECT CONTEXT	88.6 (0.21) *15*	80.0 (0.21) *15*	95.6 (0.06) *15*	81.2 (0.19) *15*
PL: NEUTRAL CONTEXT	88.6 (0.08) *15*	57.3 (0.28) *15*	91.3 (0.10) *15*	75.3 (0.23) *15*

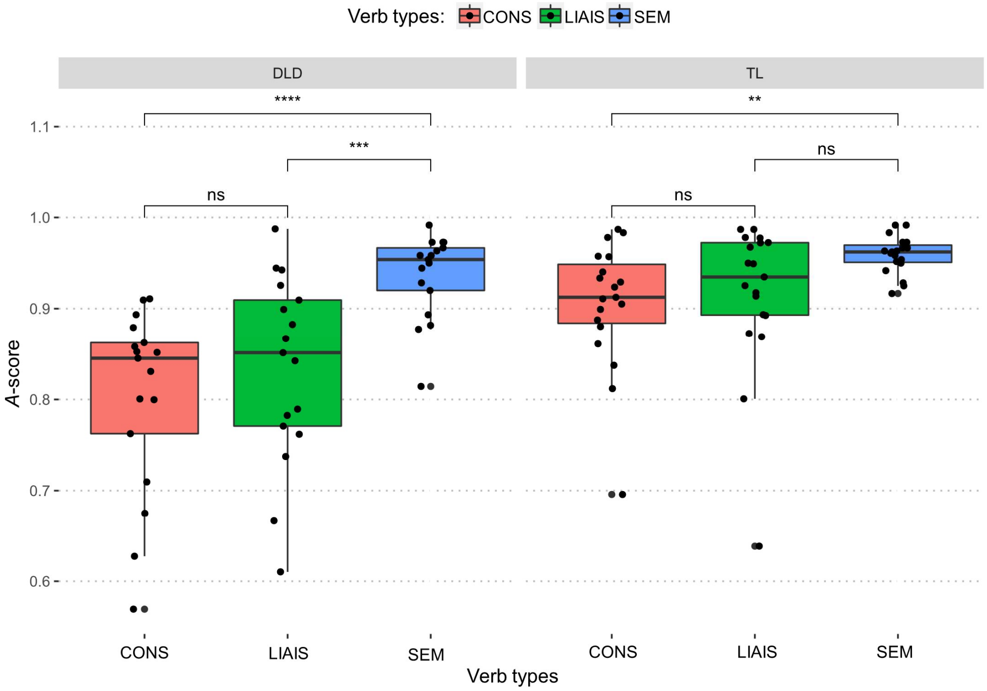

Supplementary Figure 1. Boxplots illustrating the GROUP × VERB type interaction. Notes: CONS = Consonant-final verbs; LIAIS = Liaison verbs; SEM = RegC verb Lexico-semantic condition; DLD = DLD group; TL = Typical language group. *: $p < 0.05$, **: $p < 0.01$, ***: $p < 0.001$, and ****: $p < 0.0001$

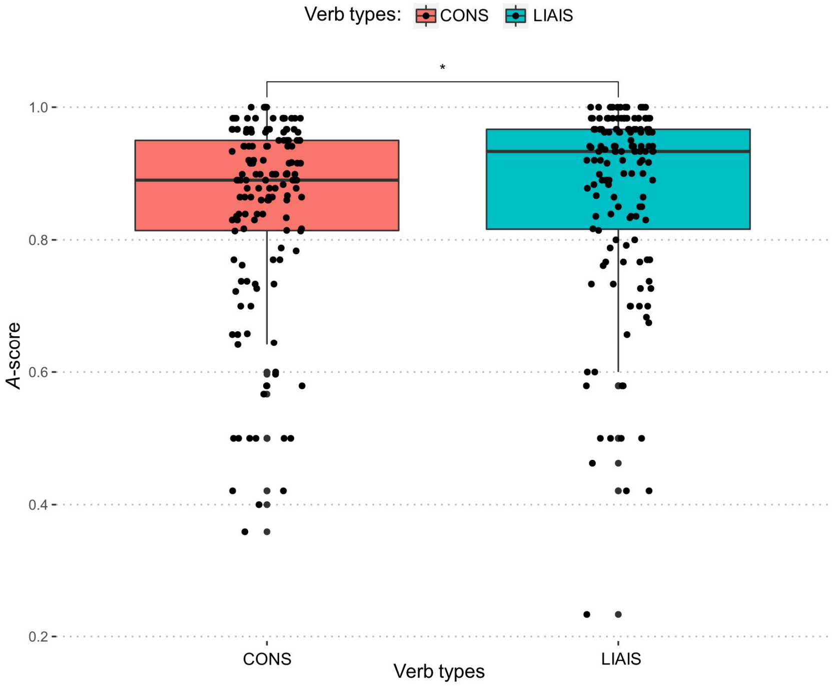

Supplementary Figure 2. Boxplots illustrating the main effect of VERB types for morphosyntactic conditions. Notes: CONS = Consonant-final verbs; LIAIS = Liaison verbs. *: $p < 0.05$

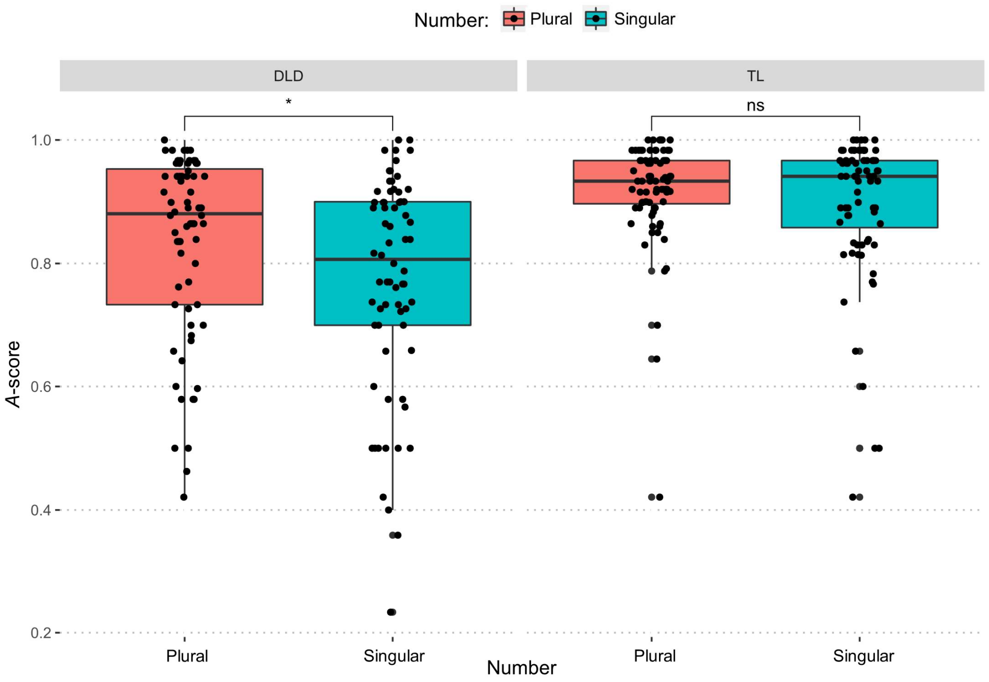

Supplementary Figure 3. Boxplots illustrating the GROUP × NUMBER interaction. Notes: DLD = DLD group; TL = Typical language group; ns = nonsignificant. *: $p < 0.05$

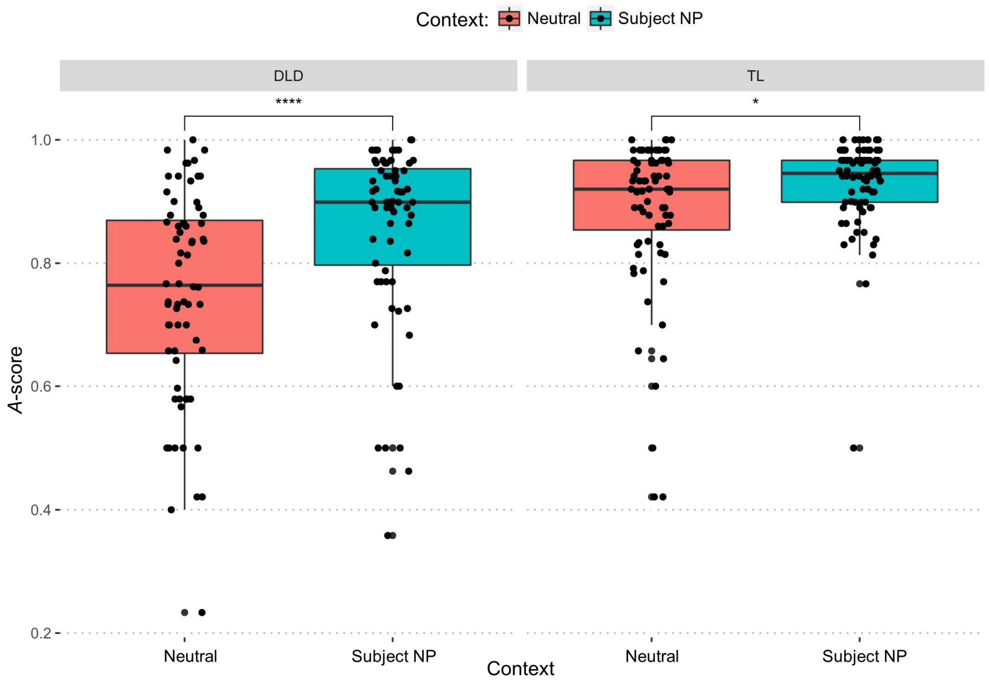

Supplementary Figure 4. Boxplots illustrating the GROUP × CONTEXT interaction. Notes: DLD = DLD group; TL = Typical language group. *: $p < 0.05$, **: $p < 0.01$, ***: $p < 0.001$, and ****: $p < 0.0001$

Section 4. Number mismatches at sentence onset

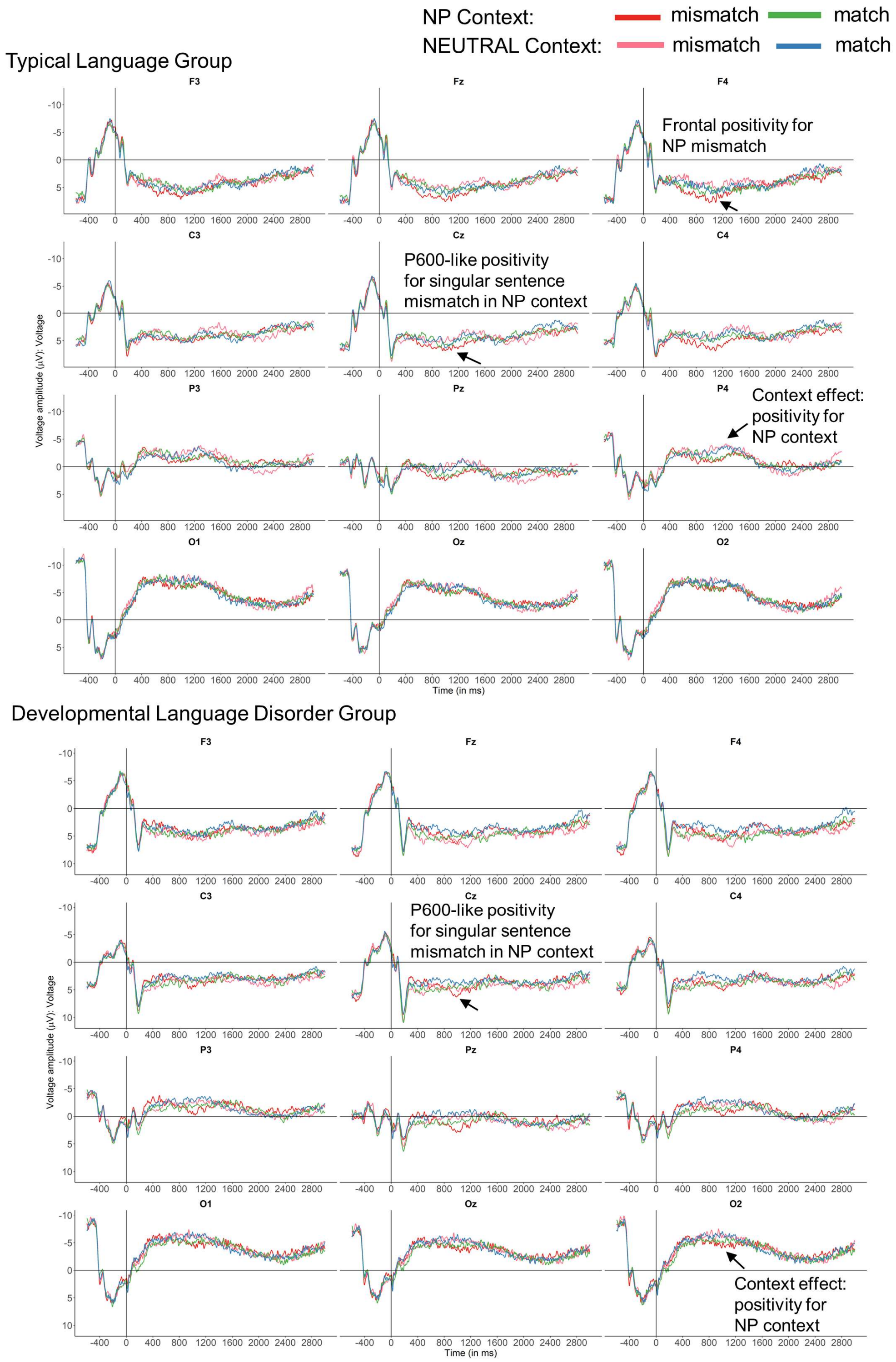

Supplementary Figure 5. ERP effects of context mismatches at sentence onset. Grand-average ERPs for the TL group (above) and the DLD group (below) are displayed at midline and eight lateral electrodes,

time-locked to sentence onset (vertical bar) using a baseline of -600 to 0 ms, and showing ERPs effect until 3000 ms. Sentence onset ERPs for both groups show that the first 900 ms (-600–300 ms) are dominated by sensory components. Visual-onset components related to picture presentation at -500 ms can be seen in posterior and occipital electrodes, and auditory-onset components elicited by the spoken sentence onset, starting at 0 ms, are elicited in fronto-central ones. When compared with the subject context correct condition (green), correct neutral contexts (blue) and mismatch (pink) conditions, the subject context mismatch condition (red) elicited a broadly distributed P600-like positivity in both groups from 600 to 1000 ms, which was driven by the singular subject context sub-conditions. From 600 to 1000 ms, both correct (green) and mismatch (red) subject contexts elicited a positivity in both groups, when compared to neutral contexts (blue, pink). This positivity migrated to more posterior and occipital regions after 1000 ms. The two neutral context conditions will be disambiguated further downstream at the verb and are not yet different at sentence onset.

Difference between groups for number mismatches at sentence onset.

Visualisation revealed a broadly distributed positivity for singular sentences in NP-subject contexts across TL and DLD groups in the 600–800 and 800–1000 ms time-windows. However, the time-course of this positivity seems different in each group: for TL participants, the positivity is largest from 600–800 ms and slightly decreases in the following time-window, whereas in the DLD group, the positivity emerges in the 600–800 ms time-window and seems to reach its maximum amplitude between 800–1000 ms. To test this assumption statistically, we ran ANOVAs comparing the two time-windows (i.e., including the additional factor TIME-WINDOW) at midline channels, on the mean amplitude of the positivity (i.e., the *difference wave* for mismatch minus match sentences). As expected, results indicated a main effect of GROUP ($F(1,33) = 4.69$, $p < 0.05$), which interacted with TIME-WINDOW ($F(1,33) = 12.45$, $p < 0.001$). First, we decomposed this interaction by GROUP, and found that in the TL group no difference was found between the two time-windows, confirming that the positivity was constant (TL: TIME-WINDOW, $F(1,18) = 2.10$, $p = 0.17$, 600–800 ms: M = 3.21μV, 800–1000 ms: M = 2.38μV). In contrast, the DLD group showed a significant TIME-WINDOW effect (DLD: $F(1,15) = 14.27$, $p < 0.001$), reflecting a substantial increase of the positivity from the first to the second time-window (600–800 ms: M = -0.23μV, 800–1000 ms: M = 1.69μV). The growing amplitude across the time-windows in the DLD group is also confirmed when decomposing by TIME-WINDOW. Indeed, we found a significant difference between groups (i.e., a larger amplitude for TL participants) in the 600–800 time-window ($F(1,33)$ =

13.36, $p < 0.0001$), but not in the 800–1000 time-window ($F(1,33) = 0.38$, $p = 0.54$). To sum up, these results showed that both groups elicited a similar positivity in response to mismatches on singular CONS verbs, but with a slower onset and a smaller amplitude for the DLD group whose P600 peaked later than in the TL group.

Overall, two effects were found in the sentence onset condition: first, a context effect in both groups, where NP-subject contexts were more positive than neutral ones. Second, a broadly distributed P600-like positivity in both groups in response to singular sentence mismatches in NP-subject contexts from 600–1000 ms. In the TL group, the positivity was strong from 600–1000 ms, and in the DLD group, it increased from 600–800 ms and peaked between 800–1000 ms.

Section 5. Noise- and artefacts-related effects for mismatches plural LIAIS verbs

We found significant interactions of CONDITION and topographical factors in lateral channels revealing effects that we interpreted as noise and eye-movements. Global ANOVAs (see Supplementary Table 7) on the 300–500 ms time-window revealed significant interactions of CONDITION and topographical factors in lateral channels, but not in midline channels, confirming that the apparent negativity in Cz for both groups was non-significant. Decomposition of HEMISPHERE × CONDITION and ANTERIORITY × HEMISPHERE × CONDITION interactions revealed a negativity in the right-hemisphere central channels channels (i.e., C4 and T8) in both groups. Focusing on C4 in Supplementary Figure 6, we can see that in the TL group this effect does not look like a typical ERP negativity, but rather noise, considering that the difference between conditions moves from positive to negative between 300–500 ms. In the DLD group, this right-lateralized negativity seemed to be part of the same polarity inversion pattern for temporal electrodes that we found in the other time-windows (1200–1500, 1500–1700) which is typical of artefacts and not of cognitive processes, as we explain in the following paragraph.

Between 1200–1500 ms, we found an effect shared by both groups as indicated by a significant HEMISPHERE × LATERALITY × CONDITION interaction in the lateral electrodes, which is subsumed by a

significant effect of CONDITION in the more lateral channels of the left hemisphere. As illustrated on the 1200–1500 ms voltage map (Supplementary Figure 6), in the DLD group a positivity was elicited in left lateral electrodes (i.e., F7, T7, P7), which explains this shared effect despite the fact that their voltage map is mainly negative. Note that the channels carrying this positivity in the DLD group are the ones that tend to be influenced by muscle movements and horizontal eye movement artifacts, which is what we believe is behind this pattern. This assumption seems to be supported by the distribution of effects on the scalp, where we observe a polarity inversion of this difference between left-anterior and right-anterior electrodes—especially F7-T7 and F8-T8, which is typical of horizonal eye-movement artefacts. Since we targeted only blinks (i.e., vertical eye movements) with the ICA artefact rejection procedure, it's possible that horizontal eye movements are contaminating these external channels. See supplementary Table 7 for decomposition of significant interactions reflecting these effects.

Supplementary Table 7. Global ANOVAs for Liaison plural verbs at verb onset, for time-windows of interest

		Negativity	Positivity	
	df	300-500	1200-1500	1500-1700
LATERAL ELECTRODES				
HEMI × COND	(1,33)	7.33**	5.75*	6.22*
HEMI R: COND	(1,34)	4.57*	—	—
HEMI L: COND	(1,34)	—	4.24*	5.13*
ANT × HEMI × COND	(2,66)	5.85*	8.13**	7.37**
FRONTAL: HEMI × COND	(1,34)	8.41**	5.93*	—
FRONTAL: RIGHT HEMI: COND	(1,34)	3.49†	—	—
FRONTAL: LEFT HEMI: COND	(1,34)	—	4.05†	—
CENTRAL: HEMI × COND	(1,34)	5.35*	10.93**	—
CENTRAL: LEFT HEMI: COND	(1,34)	—	4.11†	—
CENTRAL: RIGHT HEMI: COND	(1,34)	4.35*	—	—
LEFT HEMI: ANT × COND	(1,34)	—	—	4.30*
LEFT HEMI: FRONTAL: COND	(1,34)	—	—	7.39**
LEFT HEMI: CENTRAL: COND	(1,34)	—	—	4.49*
ANT × LAT × COND	(2,66)	5.22*	—	—
HEMI × LAT × COND	(1,33)	—	4.45*	10.73**
LATERAL: HEMI X COND	(1,34)	—	5.79*	7.61**
LATERAL: LEFT HEMI: COND	(1,34)	—	4.67*	8.87**
HEMI × ANT × LAT × COND	(2,66)	—	—	8.73**
LEFT HEMI: ANT × LAT × COND	(2,68)	—	—	3.82*
LEFT HEMI: FRONTAL × LAT × COND	(1,34)	—	—	4.57*

Left Hemi: Frontal: Lateral: Cond	(1,34)	—	—	12.00**
Group × Lat × Cond	(1,33)	—	7.70**	—
TL: Lat × Cond	(1,18)	—	6.22*	—
TL: Medial : Cond	(1,18)	—	5.21*	—
Ant × Group × Cond	(2,66)	—	—	5.16*
Frontal: Group × Cond	(1,34)	—	—	5.40*
Frontal: TL: Cond	(1,18)	—	—	5.88*
Central: Group × Cond	(1,34)	—	—	3.11†
Group × Ant × Hemi × Lat × Cond	(2, 66)	—	4.02*	7.75**
DLD: Ant × Hemi × Lat × Cond	(2,30)	—	8,17**	—
DLD: Frontal: Hemi × Lat × Cond	(1,15)	—	7.61**	—
DLD: Frontal: Left Hemi: Lat × Cond	(1,15)	—	5.28*	—
DLD: Frontal: Left Hemi: Lat: Cond			3.15†	—
DLD: Central: Hemi × Lat × Cond	(1,15)	—	8.83**	—
DLD: Central: Left Hemi: Lat × Cond	(1,15)	—	5.28*	—
DLD: Central: Left Hemi: Lat Cond	(1,15)	—	4.39†	—
Left Hemi: Ant × Lat × Group × Cond	(2,66)	—	—	5.86*
Left Hemi: Frontal: Lat × Group × Cond	(1,33)	—	—	10.09**
Left Hemi: Frontal: Medial: Group × Cond	(1,33)	—	—	4.06†
Midline electrodes				
Group × Cond	(1,33)	—	4.69*	—
TL: Cond	(1,18)	—	4.35†	—
Electrode × Group × Cond	(3,99)	—	—	3.63*
Fz: Group × Cond	(1,33)	—	—	4.69*
Fz: TL: Cond	(1,18)	—	—	4.60*

Note. Only significant results and trends are presented. Cond = Condition; Ant = Anteriority; Lat = Laterality; Hemi = Hemisphere. †: $p < 0.10$, *: $p < 0.05$, **: $p < 0.01$, and ***: $p < 0.001$.

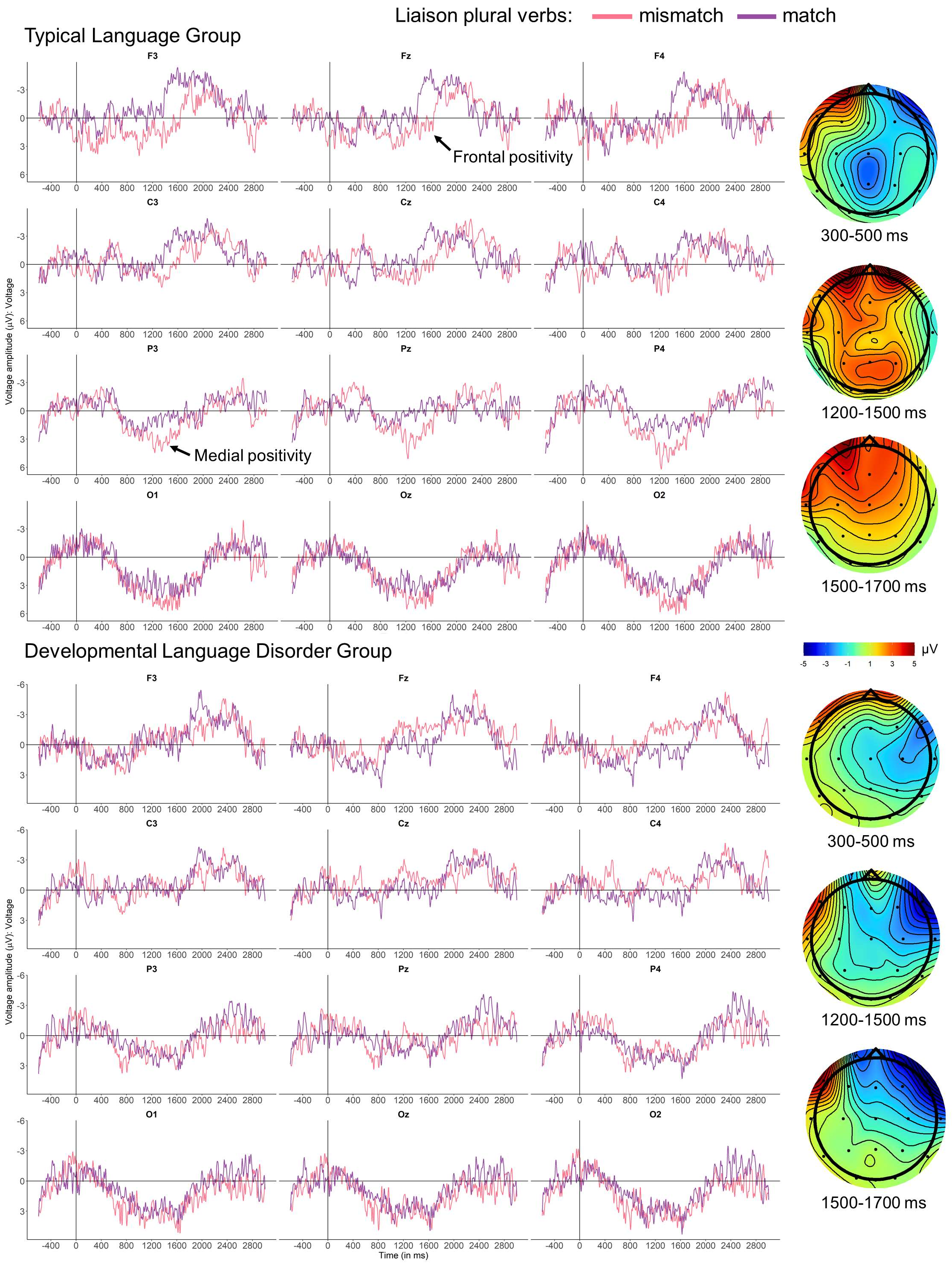

Supplementary Figure 6. ERP number mismatch effects for plural LIAIS verbs in neutral contexts. Grand-average ERPs for the TL group (above) and the DLD group (below) are displayed at midline and eight lateral electrodes, as well as voltage maps illustrating difference waves time-locked to pronoun

onset, using a baseline of -600 to 0 ms. Compared to the match condition (purple), the only reliable effects elicited by plural mismatches (pink) were found in the TL group. The TL group exhibited a small P600-like positivity from 1200–1500 ms, significant in medial electrodes and Pz, which shifted to a more frontal distribution from 1500–700 ms.

Section 6. ERPs for LIAIS singular conditions

Statistical analyses for the LIAIS singulars conditions can be found in Supplementary Table 8 and Figure 7 depicts number mismatches for singular LIAIS verbs. We observe an apparent LAN-like component at F3 in the TL group from 800–1200 ms and a small P600-like positivity in the DLD group. Based on these observations, we analysed the 800–1200 ms time-window.

Supplementary Table 8. Global ANOVAs for Liaison singular verbs at verb onset, for time-windows of interest

	df	800–1200
LATERAL ELECTRODES		
CONDITION	(1,33)	—
GROUP		—
GROUP × HEMISPHERE × CONDITION	(1,33)	5.67*
TL: LEFT HEMISPHERE ×CONDITION	(1,18)	5.35*
GROUP × ANTERIORITY × HEMISPHERE × CONDITION	(2,66)	4.86*
FRONTAL: GROUP × HEMISPHERE × CONDITION	(1,33)	6.10*
FRONTAL: TL: HEMISPHERE × CONDITION	(1,33)	7.76*
FRONTAL: TL: LEFT HEMISPHERE: CONDITION	(1,18)	6.56*
GROUP × LATERALITY × HEMISPHERE × CONDITION	(1,33)	4.95*
LATERAL: GROUP × HEMISPHERE × CONDITION	(1,33)	6.06*
LATERAL: TL: HEMISPHERE × CONDITION	(1,18)	4.30 †
GROUP × LATERALITY × ANTERIORITY × HEMISPHERE × CONDITION	(2,66)	6.00*
TL: LATERALITY × ANTERIORITY × HEMISPHERE × CONDITION	(2,36)	4.30*
TL: LATERAL: ANTERIORITY × HEMISPHERE × CONDITION	(2,36)	5.39*
TL: LATERAL: FRONTAL: HEMISPHERE × CONDITION	(1,18)	6.06*
TL: LATERAL: FRONTAL: LEFT HEMISPHERE: CONDITION	(1,18)	13.37*
MIDLINE ELECTRODES		
CONDITION	(1,33)	—
GROUP	(1,33)	—
GROUP × ELECTRODE × CONDITION	(1,99)	—

Note. Only significant results and trends are presented. †: $p < 0.10$, *: $p < 0.05$, **: $p < 0.01$, and ***: $p < 0.001$.

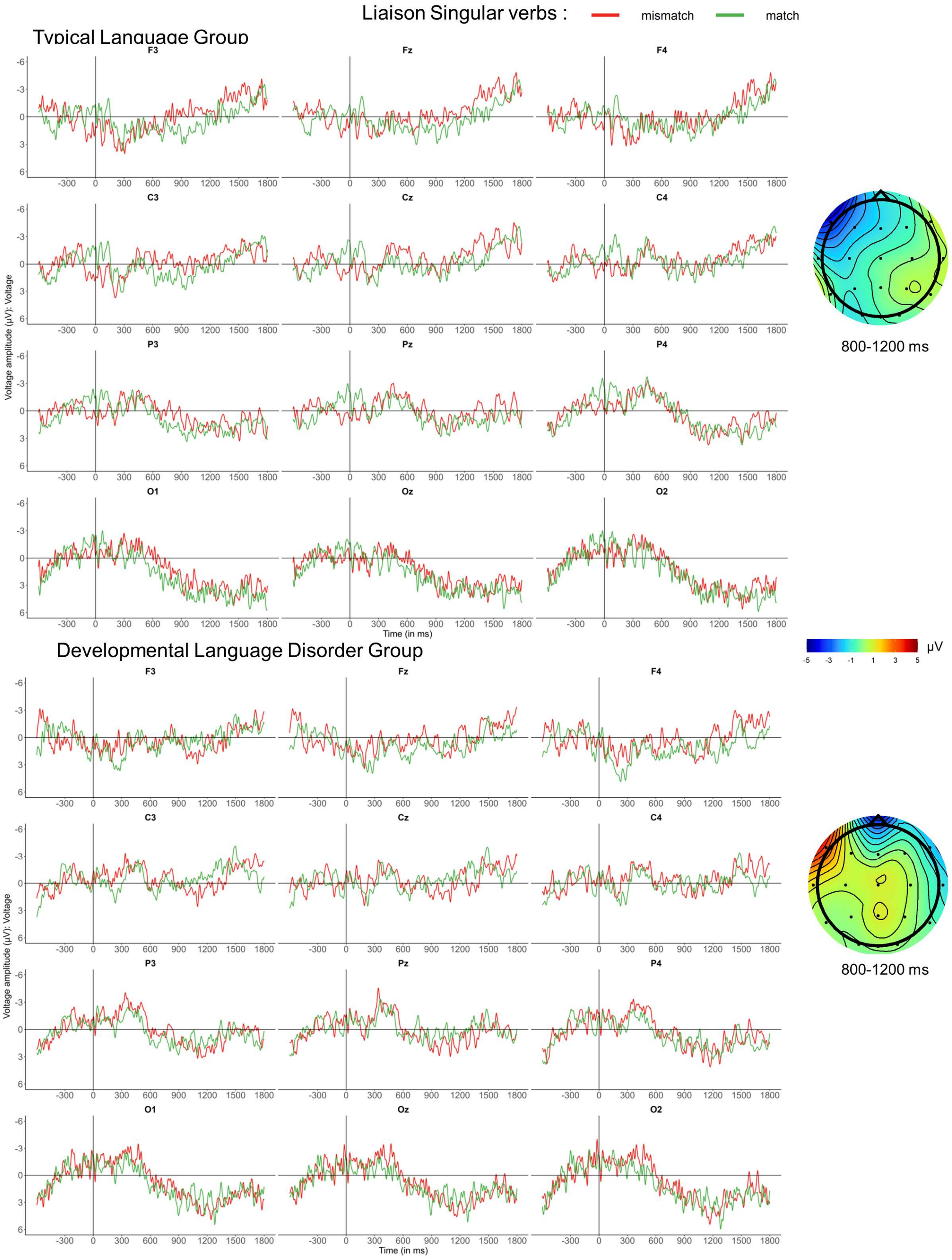

Supplementary Figure 7. ERP number mismatch effects for singular LIAIS verbs in neutral contexts. Grand-average ERPs for the TL group (above) and the DLD group (below) are displayed at midline and eight lateral electrodes, as well as voltage maps illustrating difference waves, time-locked to pronoun

onset (vertical bar) using a baseline of -600 to 0 ms. Compared to the match condition (green), plural mismatches (red) did not elicit any consistent ERP pattern in either group.

The global ANOVA (see Supplementary Table 8) revealed effects in the TL group only, as supported by significant interactions involving topographical factors, GROUP and CONDITION. This LAN-like negativity was significant only in the more lateral electrodes, namely F7, and was driven by one participant. Since F7 is more prone to be influenced by eye movements, we judged this effect to be unreliable. Overall, our analyses did not point to any consistent ERP pattern for singular LIAIS verb mismatches in both groups. This is not surprising considering that LIAIS singular verb mismatches did not induce significant effects in the adult's group as well (see discussion in Courteau et al., [10] for details on why this may be).

Section 7. Analyses of the positivity in Consonant-Final singular conditions

In a nutshell, the CONS verb singular mismatches elicited a significant positivity broadly distributed for the TL group from 1000–1400 ms and a significant positivity in frontal channels of the right hemisphere for all participants from 1400–1800 ms. These results raised the possibility that the two positivities from 1000–1800 ms reflected the same ERP component, but with an earlier onset for the TL group (1000 ms) versus the DLD group (1400 ms). We ran additional ANOVAs directly comparing the two time-windows, see Supplementary Table 9. As expected, TIME-WINDOW showed significant interactions with GROUP and topographical factors, confirming that the P600-like positivity from 1000–1400 ms was elicited in the TL group only, and was a different ERP component from the positivity in the 1400–1800 time-window.

Supplementary Table 9. Global ANOVAs for Consonant-Final singular verbs comparing directly the 1000–1400 and 1400–1800 ms time-windows.

	df	
LATERAL ELECTRODES		
CONDITION	(1,33)	5.64*
TIME WINDOW (TW)	(1,33)	4.81*
TW × GROUP × COND		3.88 †
TW × HEMI × COND	(1,33)	6.83*

TW × ANT × HEMI × COND	(2,66)	6.75**
1400-1800: ANT × HEMI × COND	(1,34)	4.96*
1400-1800: FRONTAL: HEMI X COND	(1,34)	4.85*
1400-1800: FRONTAL: RIGHT HEMI: COND	(1,34)	11.2**
TW × LAT × HEMI × COND	(1,33)	5.60*
LATERAL: TW × HEM × COND	(1,34)	6.90**
LATERAL: RIGHT HEMI: TW × COND	(1,34)	3.32 †
TW × ANT × LAT × HEM × COND	(2,66)	6.60**
FRONTAL: TW × LAT × HEM × COND	(1,34)	7.78**
FRONTAL: LEFT HEMI: TW × LAT × COND	(1,34)	10.29**
FRONTAL: LEFT HEMI: LAT: TW × COND	(1,34)	3.25†
TW × GROUP × LAT × COND	(1,33)	6.97*
TL: TW × LAT × COND	(1,18)	4.11†
DLD: TW × LAT × COND	(1,15)	3.66†
GROUP × HEMI × LAT × COND	(1,33)	4.58*
MIDLINE ELECTRODES		
CONDITION	(1,4)	5.85*
TIME WINDOW	(1,33)	—
TIME WINDOW × GROUP × COND		5.33*
1000-1800: GROUP × COND	(1,33)	—
1000-1400: GROUP × COND	(1,33)	7.42**
1000-1400: DLD: COND	(1,15)	—
1000-1400: TL: COND	(1,18)	7.30*

Note. Only significant results and trends are presented. Cond = Condition; Ant = Anteriority; Lat = Laterality; Hemi = Hemisphere. †: $p < 0.10$, *: $p < 0.05$, **: $p < 0.01$, and ***: $p < 0.001$.

References

[1] Pourquié M. fLEX: Multilingual assessment of inFlectional and LEXical processing. Software, Intellectual Property 2016-01 2015;88.

[2] New B, Pallier C, Ferrand L, Matos R. Une base de données lexicales du français contemporain sur internet: LEXIQUE™//A lexical database for contemporary french: LEXIQUE™. L'année Psychologique 2001;101:447–62. https://doi.org/10.3406/psy.2001.1341.

[3] Huang Y, Ferreira F. The Application of Signal Detection Theory to Acceptability Judgments. Front Psychol 2020;11. https://doi.org/10.3389/fpsyg.2020.00073.

[4] Zhang J, Mueller ST. A note on ROC analysis and non-parametric estimate of sensitivity. Psychometrika 2005;70:1–10. https://doi.org/10.1007/s11336-003-1119-8.

[5] Macmillan NA, Creelman CD. Detection theory: A user's guide, 2nd ed. Mahwah, NJ, US: Lawrence Erlbaum Associates Publishers; 2005.

[6] Lachaud CM, Renaud O. A tutorial for analyzing human reaction times: How to filter data, manage missing values, and choose a statistical model. Applied Psycholinguistics 2011;32:389–416.

[7] Wobbrock JO, Findlater L, Gergle D, Higgins JJ. The aligned rank transform for nonparametric factorial analyses using only anova procedures. Proceedings of the SIGCHI Conference on Human Factors in Computing Systems, New York, NY, USA: Association for Computing Machinery; 2011, p. 143–6. https://doi.org/10.1145/1978942.1978963.

[8] Cohen J. A power primer. Psychological Bulletin 1992;112:155.

[9] Cohen J. Statistical power analysis for the behavioral sciences. 2nd ed. Hillsdale, N.J: L. Erlbaum Associates; 1988.

[10] Courteau É, Martignetti L, Royle P, Steinhauer K. Eliciting ERP components for morphosyntactic agreement mismatches in perfectly grammatical sentences. Front Psychol 2019;10. https://doi.org/10.3389/fpsyg.2019.01152.
